# Introduction and Extension of the Unified Theory of Multicenter Bonding: The Role of the Charge-Shift Bonding

**DOI:** 10.3390/molecules31010082

**Published:** 2025-12-24

**Authors:** Francisco Javier Manjón, Hussien H. Osman, Álvaro Lobato, Fernando Izquierdo-Ruiz, Enrico Bandiello, Samuel Gallego-Parra, Ángel Vegas, Matteo Savastano, Alfonso Muñoz

**Affiliations:** 1 Instituto de Diseño para la Fabricación y Producción Automatizada, MALTA Consolider Team, Universitat Politècnica de València, 46022 Valencia, Spain or hussien.helmy@uv.es (H.H.O.); ebandie@upv.es (E.B.); sagalpar@upvnet.upv.es (S.G.-P.); 2 Instituto de Ciencia de los Materiales de la Universitat de València, MALTA Consolider Team, Universitat de València, 46100 Valencia, Spain; 3 Chemistry Department, Faculty of Science, Helwan University, Cairo 11795, Egypt; 4 Departamento de Química Física, MALTA Consolider Team, Universidad Complutense de Madrid, 28040 Madrid, Spain; a.lobato@ucm.es (Á.L.); ferizqui@ucm.es (F.I.-R.); 5 Independent Researcher, Calle Espinarejo 1, 28400 Collado Villalba, Spain; diegas2002@gmail.com; 6 Department of Human Sciences for the Promotion of Quality of Life, University San Raffaele Roma, Via di Val Cannuta 247, 00166 Rome, Italy; matteo.savastano@uniroma5.it; 7 Departamento de Física, MALTA Consolider Team, Universidad de La Laguna, 38205 La Laguna, Spain; amunoz@ull.edu.es

**Keywords:** classical bonding, unconventional bonding, charge-shift bonding, electron-rich multicenter bonding, electron-deficient multicenter bonding

## Abstract

Classical chemical bonding is typically categorized into primary, strong interactions, such as covalent, ionic, and metallic bonds, and secondary, weak interactions, such as van der Waals forces, the hydrogen bond, and their likes (halogen bond, chalcogen bond, etc.). However, other not-so-known bonding mechanisms also play a crucial role in chemical systems. Particularly important are the charge-shift bond (CSB) and the multicenter bonds, i.e., the electron-rich multicenter bond (ERMB), also known as hypervalent or three-center-four-electron (3c-4e) bond, and the electron-deficient multicenter bond (EDMB), also known as the three-center-two-electron (3c-2e) bond in molecules and, more recently, as the two-center-one-electron (2c-1e) bond in extended solids. We consider that these latter interactions have not yet received the proper attention of the scientific community, even though multicenter interactions were proposed in the early days of Quantum Mechanics. In this work, we aim at providing: (i) a concise historical overview of the two types of multicenter bonds; (ii) a short introduction to the recently proposed unified theory of multicenter bonding (UTMB), which elucidates the origin and mechanisms of formation of both ERMBs and EDMBs; and (iii) an extension of the UTMB to include CSBs, due to the strong relationship between ERMBs and CSBs. We hope that the integrated perspective of chemical bonding, the heartland of chemistry, offered by the UTMB (beyond traditional and historical assumptions) will help researchers to understand materials properties and will provide a framework allowing the development of advanced materials for enhanced technological applications.

## 1. General Introduction

In contrast to conventional covalent, ionic, and polar covalent bonds, in which electrons are fully localized and shared/transferred between two centers, and to metallic bonds, with fully delocalized electrons shared among many centers, a multicenter bond is a chemical bond between more than two centers, i.e., extended along three or more centers. Therefore, a multicenter bond can be considered as a special concatenation or combination of two-center bonds, different from the concatenation of covalent or ionic bonds, where electrons are partially delocalized along the directions of the three or more bonded atoms, unlike what happens in covalent or ionic bonds. Two kinds of multicenter bonds have been known since 1950: the electron-rich multicenter bond (ERMB) and the electron-deficient multicenter bond (EDMB).

**Figure 1:**
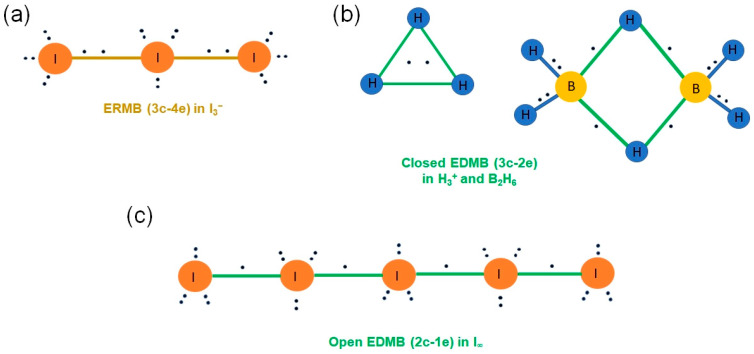
(**a**) ERMB (3c-4e bond) present in the I_3_^−^ anion. (**b**) EDMB (3c-2e bond) present in the H_3_^+^ cation and B_2_H_6_ molecule. (**c**) EDMB (2c-1e bond) present in the infinite linear chain of I atoms, I_∞_. Atoms, bonds, and electrons are represented as filled circles, solid bars, and black dots, respectively. The two-center single covalent bonds are shown in blue color, while the two-center bonds forming part of ERMBs and EDMBs are shown in gold and green color, respectively. Electrons located in the center of the bonds indicate that they are shared by the two atoms, while electrons located close to one atom indicate that they are not totally shared.

Throughout the 20th century, these two types of multicenter bonds were well studied in molecules, but not so well in solids. This limitation has precluded a complete understanding of the two multicenter bonds until now. In the literature, the most studied ERMB was the three-center-four-electron (3c-4e) bond (present in isoelectronic XeF_2_ and I_3_^−^, see Figure 1a), while the most studied EDMB was the three-center-two-electron (3c-2e) bond (present in H_3_^+^ and B_2_H_6_, see Figure 1b). The large number of studies on molecules led to the belief that EDMBs could only be possible in valence electron-deficient elements (elements from groups 1, 2, 11, 12, and 13), while ERMBs could only be possible in valence electron-precise and electron-rich elements (elements of group 14 and from group 15 to 18, respectively) [1]. Notice that the definition of a valence electron-deficient, electron-precise, and electron-rich element is related to the ratio between the number of valence electrons available for bonding and the number of covalent bonds that are needed for the atoms to reach the noble gas configuration, i.e., to satisfy the doublet/octet rule of Lewis-Langmuir [2,3].

In this context, a couple of comprehensive articles have recently been published presenting the Unified Theory of Multicenter Bonding (UTMB) [4,5]. The UTMB addresses the origin and formation mechanism of ERMBs and EDMBs and emphasizes that the formation of multicenter bonds is favored at high electronic density conditions, i.e., when there is a high orbital overlap of electronic clouds, as it occurs: (i) in strongly compressed matter (at high pressure); (ii) when atoms in a group are substituted by their heavier analogs; and (iii) when systems are reduced (i.e., systems gain electrons).

In brief, the UTMB states that both multicenter bonds can be formed from original primary two-center bonds and additional secondary two-center bonds (or interactions) following a three-stage process. On one hand, ERMBs typically form from a primary bond and a secondary interaction, resulting in a linear or quasi-linear 3c-4e bond, i.e., a three-center bond or trimer in which there are two electrons between every two atoms; however, these electrons are not fully shared as in a simple covalent bond. In this configuration, the central atom of the trimer is hypercoordinated, and the multicenter character of the 3c-4e bond is due to the presence of delocalized electrons along the two collinear or quasi-collinear two-center bonds that form the three-center bond. In the process of ERBM formation, some non-bonding electrons, present as lone electron pairs (LEPs) in the secondary bond, get transformed into bonding electrons when the ERMB is formed, so that the original LEP disappears. In this process, the primary bond is usually enlarged and its electronic charge redistributed so that the central atom does not violate the doublet/octet rule. Therefore, the central atom of the trimer is hypercoordinated but not hypervalent. In fact, the UTMB establishes that linear or quasi-linear ERMBs cannot be larger than three centers; otherwise, the doublet/octet rule is violated for the central atoms of the linear molecule. In other words, ERMBs are restricted to linear or quasi-linear 3c-4e bonds because, in this configuration, the central atom does not violate the doublet/octet rule. 

On the other hand, EDMBs typically form from a primary two-center bond and a secondary two-center interaction, resulting in a 3c-2e bond, i.e., a three-center bond or trimer in which there is basically one electron between every two atoms, so that it can be considered as formed by two concatenated two-center-one-electron (2c-1e) bonds. Similar to the ERMB, the central atom of the trimer in a 3c-2e EDMB is usually hypercoordinated, and the multicenter character of this bond is due to the presence of delocalized electrons along the two two-center bonds that form the three-center bond. The formation of trimers with 3c-2e bonds is typical of electron-deficient elements, in which different geometries (from angular to linear) have been found; however, the situation seems to be different when EDMBs are formed by electron-rich elements in solids. In this case, linear or quasi-linear concatenated 3c-2e bonds or, equivalently, concatenated 2c-1e bonds are observed forming infinite atomic chains along certain directions. In this case, all atoms in the chain are hypercoordinated, and the multicenter character of this bond is due to the electron delocalization along the chain, which leads to a considerable electrical conductivity along the chain. In the process of EDMB formation, the charge for the formation of the 3c-2e bonds (either isolated or concatenated in a linear chain) mainly comes from the primary bond; i.e., one electron of the typical primary two-electron bond is transferred to the interatomic space of the secondary non-covalent bond so that, at the end, both primary and secondary bonds form two equal or almost equal 2c-1e bonds (there is no longer a primary and a secondary bond). In this process, the primary bond is usually enlarged as it loses charge, and no atom in the 3c-2e bond (either isolated or concatenated) violates the doublet/octet rule. Therefore, atoms in EDMBs can also be hypercoordinated but not hypervalent. For this reason, we will not use the word “hypervalent” in our description of multicenter bonds hereon.

The UTMB has challenged the current understanding of chemical bonding, much like the discovery of the charge-shift bond (CSB) [6], since it has shown that linear EDMBs, in the form of 2c-1e bonds, can also occur for electron-precise (Si) and electron-rich (As, Po) elements, unlike previously assumed [1]. Some of the UTMB claims, such as the presence of multicenter character in 2c-1e bonds and the limitation of ERMBs to linear 3c-4e bonds, seem to be supported by recent calculations in phase change materials (PCMs) [7]. We want to mention that the bond classification provided by the UTMB is based on the number of electrons shared (ES) and transferred (ET) between any two atoms, the doublet/octet rule, and the valence shell electron pair repulsion (VSEPR) theory [8].

With the three above-mentioned ideas, the UTMB has explained the chemical bonding in different materials of interest in many diverse fields. Initial efforts of the UTMB have been directed to demonstrate that EDMBs exist in electron-precise and electron-rich elements, thus providing a unified view of ERMBs and EDMBs in these elements. This has allowed us to explain: (i) the presence of EDMBs in the two known phases of elemental polonium at ambient pressure as well as the high-pressure sixfold-coordinated phases of pnictogens (As, Sb, Bi) and chalcogens (Se, Te) [4], in the crystalline phases of PCMs (SnTe, PbS, PbSe, and PbTe) at room pressure [9,10], and in related ternary compounds, such as PbBi_2_Te_4_, at room pressure [11]; (ii) the different multicenter bonds present in several Zintl compounds (Li_2_Sb, BaZnSb_2_, Cs_2_Te_5_, Cs_2_TeI_6_, and TlTe) [5]; (iii) the EDMBs that tend to be formed in hypercoordinated I atoms in metal iodates [12,13]; (iv) the multicenter bonds present in Rashba semiconductors BiTeX (X = Cl, Br, I) at room pressure [14]; and (v) the EDMBs present in pressure-induced AIO_3_ perovskites, like RbIO_3_ and CsIO_3_ [15].

Moreover, the UTMB has allowed us: (i) to propose (for the first time) a mechanism for the formation of the perovskite structure in BX_3_ and ABX_3_ compounds as the result of a polymerization process of the BX_3_ units [15]; (ii) to explain the different geometries found (and why others have not been found) in polyiodides, an issue in supramolecular chemistry for more than 50 years [16]; (iii) to show (for the first time) the transformation of ERMBs in EDMBs in compressed AX_3_ halides [17]; and (iv) to account for the chemical bonding present in an infinite linear chain of H and I atoms [5,17,18]. Finally, the UTMB has challenged the current understanding of metallic bonding since a multicenter character along certain directions has recently been discovered in the metallic bonds of elements where *p*-type electrons participate in bonding [19].

**Figure 2:**
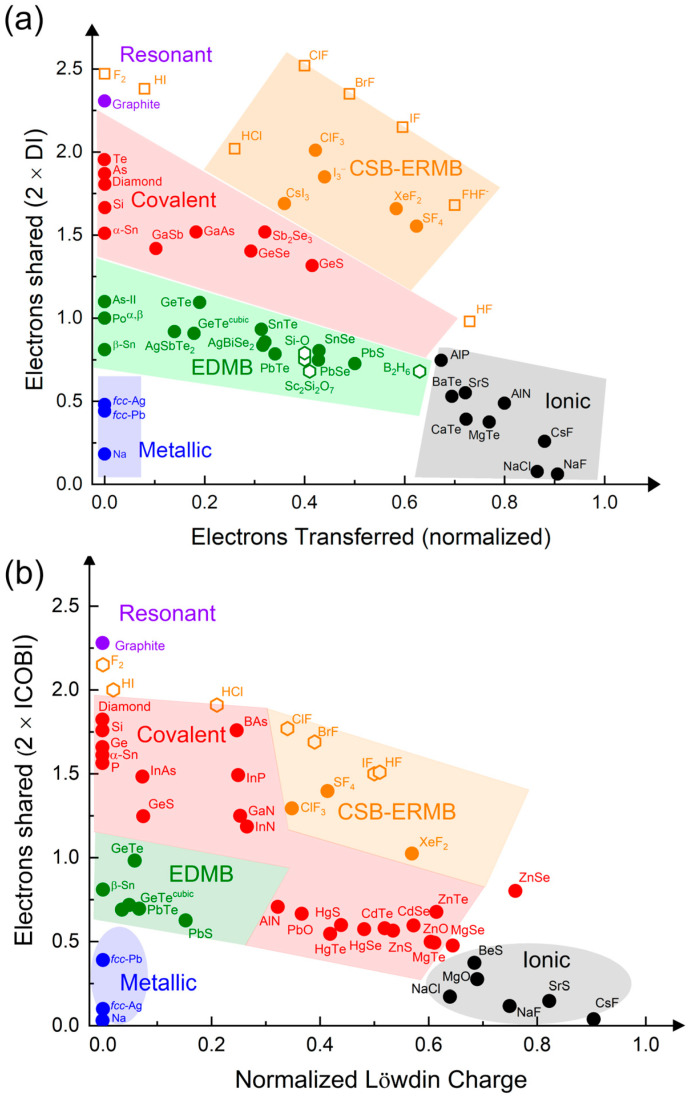
(**a**) ES vs. ET (normalized) map obtained from density-based calculations. The ES value is obtained as twice the delocalization index and the normalized ET value is obtained from Bader’s atomic charges. (**b**) ES vs. ET (normalized) map obtained from orbital-based calculations. The ES value is obtained as twice the ICOBI(2c) and the normalized ET value is obtained from Löwdin’s atomic charges. Resonant, covalent, ionic, and metallic bonds are represented in purple, red, black, and blue colors, respectively. In addition, EDMBs are represented in green color, while CSBs and ERMBs are represented in orange color. Some of the EDMBs, ERMBs, and CSBs commented in this work are plotted. Open hexagons and filled circles represent the results obtained in this work and values derived from previous studies [4,5,10,19], respectively.

Setting aside these astonishing results for a moment, the UTMB offers two particularly significant contributions to the holistic understanding of chemical bonding. Firstly, it demonstrates that EDMBs exist in electron-precise elements (tetrels) and in electron-rich elements (pnictogens, chalcogens, and halogens), bringing for the first time a unified view of ERMBs and EDMBs, as already mentioned. Secondly, it provides a more comprehensive and unitary view of chemical bonding, as it places the two multicenter bonds on equal footing with the classical resonant, covalent, ionic, and metallic bonds. This can be visualized by plotting all these bonds in a two-dimensional map showing ES vs. ET (normalized) (Figure 2). The different bonding types are located at different but adjacent regions of the map [5]. We wish to underline that the 2D ES vs. ET map can be considered as a new version of the classic covalency vs. ionicity diagrams traditionally used to plot and classify chemical bonds. The advantage of this new plot is that the ES and ET parameters can be directly computed from density functional theory (DFT) simulations, using either density-based or orbital-based analysis [20].

In this paper, we firstly provide a concise historical overview of chemical bonding, with emphasis on the evolution of the multicenter bond concept and its relationship with classical resonant, covalent, ionic, and metallic bonds, and the more recent CSB. Afterwards, we explain the origins of the UTMB and its relation with the high-pressure studies that have allowed us to put an end to the dispute between the metavalent and hypervalent bonding models in the crystalline phases of PCMs [4,9,10]. As will be seen later, a simple model of a generic atomic chain with two different bonds (one strong and another weak) is useful to understand the formation of EDMBs under high-pressure conditions. Finally, we introduce the UTMB by considering well-known concepts in chemical literature, like the classical bonds, the doublet/octet rule, the VSEPR theory, and the 8–N rule (also known as Pearson’s rule and valid for group 14 to 18 elements) [21], which predicts the number of covalent bonds to be established by atoms with a total number N of valence *s* and *p* electrons (to satisfy the octet rule). In the last section, we show that the formation of EDMBs and ERMBs can be understood as an insertion of atoms inside covalent and CSBs, and also propose an extension of the UTMB to include CSBs, exploiting the parallelism of chemical bonding between CSBs and ERMBs.

## 2. A Historical Note on Chemical Bonding: Multicenter Bonds

**Figure 3:**
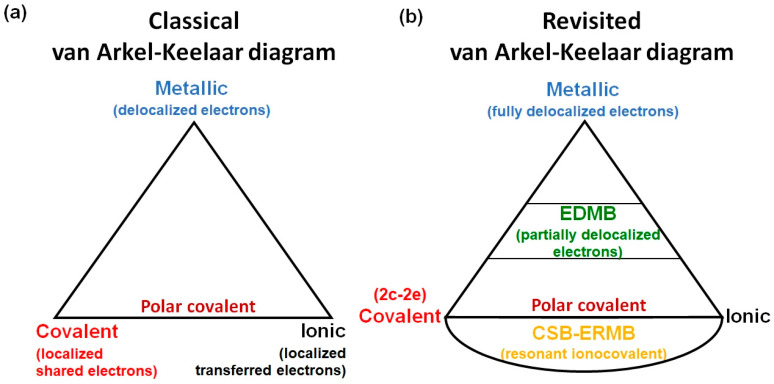
(**a**) Classical van Arkel–Ketelaar diagram. (**b**) Revisited van Arkel–Ketelaar diagram.

Chemistry textbooks provide a classical picture of chemical bonding, which includes, on one hand, primary strong interactions, such as covalent, ionic, and metallic bonds, and on the other hand, secondary weak interactions, such as van der Waals interactions, the hydrogen bond, and their likes (halogen bond, chalcogen bond, etc.). In particular, primary strong bonds are plotted in the classical van Arkel–Ketelaar diagram (Figure 3a). Covalent and ionic bonds represent situations where electrons are fully localized in the space between two atoms, and the metallic bond represents a situation where electrons are fully delocalized among the atoms that compose the system, so that electrons behave almost as a free electron gas (Fermi gas). In a pure covalent bond, also known as a two-center-two-electron (2c-2e) bond, the two localized electrons can be considered as fully shared between two atoms; i.e., they are considered as shared-shell (SS) interactions, while in a pure ionic bond, electrons can be considered as fully transferred from the most electropositive atom to the most electronegative atom, and are considered as closed-shell (CS) interactions. The most common case was usually considered a mixture of both extremes, i.e., the polar covalent bond. In addition, we should mention that the covalent picture includes multiple (double and triple) covalent bonds as well as delocalized resonant bonds, which are typically a mixture of single and double covalent bonds.

On the other hand, regarding secondary interactions, the weakest bonds of this kind are traditionally considered to be CS interactions with a negligible overlap of the clouds of valence electrons; i.e., they are considered pure electrostatic interactions in which quantum effects do not play any significant role. However, the strongest secondary interactions, like strong hydrogen bonds, are known to exhibit considerable SS character [22,23,24].

In this context, it must be noted that other types of exotic bonds do occur in nature that are not contemplated in the classical picture of chemical bonding. We can cite the multicenter bonds, which are bonds extended to more than two centers, and the (two-center) CSB [25]. We will comment on the CSB in the last section, so now we will pay attention to the multicenter bonds, which are intermediate-strength bonds that can be considered between the primary strong bonds and the secondary weak interactions, i.e., between the covalent SS interactions and the non-covalent CS interactions [22]. Interestingly, multicenter bonds were already proposed in the early days of Quantum Mechanics, as “Mehrzentrensystem” in the works of F. Hund, F. Bloch, and E. Hückel [26,27,28]. Later on, two types of multicenter bonds—also referred in the literature as multicentre, multi-center, or multi-centre—were defined around 1950: EDMBs [29,30,31,32] and ERMBs [33,34]. Seminal works defined the simplest EDMBs as 3c-2e bonds and the simplest ERMBs as 3c-4e bonds. These bonds were clearly considered different from simple covalent 2c-2e bonds.

In the late 1960s, Musher published a review of molecules showing ERMBs [35], including linear trimers, like XeF_2_ and I_3_^−^, and introduced the term “hypervalency” to emphasize that the central atom of these trimers was surrounded by more than eight electrons, thus suggesting a violation of the octet rule. As a result of Musher’s work, ERMBs were subsequently known either as hypervalent bonds or hyperbonds. These two terms have proved to be so intuitive and persuasive that the terms “ERMBs” and “3c-4e bonds” have been almost abandoned, and these bonds are known in modern literature as hyperbonds. In this context, we want to stress that the term “hypervalency” aimed to highlight that there were atoms with more bonds than those predicted by the 8–N rule. Therefore, the term “hypervalency” was indeed related to the presence of hypercoordinated atoms, as if those atoms would have a larger valency than expected; however, the octet rule is violated only if one considers that all the bonds are simple covalent 2c-2e bonds, which is not the case, as discussed in the UTMB [5]. We have already discussed in the UTMB (ref. [5]) that the term “hypervalent” should be discarded in favor of the term “hypercoordinated”, since the atoms involved in “hypervalent” molecules do not show a violation either of the 8–N rule or of the doublet/octet rule.

It must be mentioned that several reviews have been published regarding molecules and solids with EDMBs and ERMBs [36,37,38,39,40]. Some of them have given rise to the “increased-valence” theory related to ERMBs. That theory explains the formation of the ERMB as due to the transformation of the non-bonding electrons of a LEP into bonding electrons, i.e., the donation of the two electrons of the LEP of one atom to a neighbor atom to form a dative bond with it [37,39,40]. This explanation of donor-acceptor (D-A) interactions in molecules is indeed accepted by the IUPAC to also explain Lewis acid–base reactions [41,42,43] and agrees with the view of formation of ERMBs included in the UTMB [5]. Precisely, the study of D-A interactions is another underexplored confluence point since these chemical interactions have been mainly studied in molecules and not in solids, in the same way as previously commented for the case of multicenter bonds. As an example, we can cite both Gutmann’s work on the quantification of Lewis acidity/basicity via donor and acceptor numbers [44] and its starting point, the so-called Gutmann’s rules [45]. These rules will be commented on in the last section in relation to the formation of ERMBs and EDMBs, although Gutmann was not aware of the relationship between D-A interactions and multicenter bonds. Finally, the more recent review by Papoian and Hoffman on hypervalent molecules and solids provided a comprehensive and elegant electron-counting rule for the formation of multicenter bonds [38], which inspired the electron-counting rule proposed in the UTMB [5].

Noteworthy, W. Lipscomb was awarded the Nobel Prize in Chemistry in 1976 for the explanation of the geometries and properties of boranes (boron hydrides, B_x_H_y_) with the use of ionocovalent bonds and EDMBs [46]. Later, W. A. Harrison and coworkers developed a tight-binding theory of multicenter bond in the 1980s to account for the electronic band structure of solids whose bonds cannot be described with *sp*^3^ hybrids, i.e., solids with atoms showing more than four interatomic bonds, thus violating the 8–N rule for covalent bonding [47]. Despite these important antecedents, materials researchers inadvertently use in most of their works the terms “covalent”, “ionic”, and “metallic” to describe bonds in materials that indeed exhibit one of the two types of multicenter bonds. One of the aims of the proponents of the UTMB is to solve this issue.

In summary, much of the current understanding of EDMBs and ERMBs comes from the study of molecules, being solids largely unexplored in this realm. In this context, it was assumed that EDMBs occur in molecules and materials with valence electron-deficient elements, such as the hydrogenonium cation H^3+^, elemental boron, boranes (especially the most elemental one, the diborane B_2_H_6_), and BaAl_4_, among others. On the other hand, it was assumed that ERMBs are typically present in molecules and materials with valence electron-rich elements, such as molecules I_3_^−^, XeF_2_, XeF_4_, XeF_6_, simple cubic (sc) Sb, and several Zintl phases, such as Li_2_Sb, BaZnSb_2_, and TlTe [1,38]. Finally, valence electron-precise elements could be considered to be located in a no-man’s land and could participate in EDMBs or ERMBs depending on the electronic configuration of the system.

As already mentioned, the understanding of chemical bonds exposed in the previous paragraph has been challenged by the UTMB, once it was proved that EDMBs can occur in valence electron-rich elements [4,5]. However, before getting into the UTMB with more detail, it must be stressed that, even before the appearance of the UTMB, several criticisms have been reported regarding the chemical bonding picture summarized in the previous paragraph. First, the very existence of ERMBs was questioned in several works arguing the lack of enough evidence for considering the ERMB as an independent type of chemical bond [48,49]. Second, it is well known that the hydrogen element occupies a unique, sometimes misleading, position in the periodic table. On one hand, despite being located far to the left, it cannot be considered as a valence electron-deficient element, but as a valence electron-precise element, like group-14 elements. Note that H has the same number of valence electrons (1) as the number of bonds required to form covalent (2c-2e) bonds (1) according to the 2–N rule for atomic coordination (the special 8–N rule for hydrogen), being N is the total number of valence *s*-type electrons. Therefore, from the point of view of covalent bonding, H is equivalent to tetrels, which have the same number of valence electrons (4), as the number of bonds required to form 2c-2e bonds (4) according to the 8–N rule. On the other hand, hydrogen is obviously nothing like alkali metals and can, to some extent, be thought of as a pseudo-halogen, in the sense of being 1 valence electron away from a noble gas configuration. In this respect, it is noteworthy the prominence of both hydrogen and halogen bonds in classical and contemporary supramolecular chemistry. We remind how, at least the hydrogen bond, is known to be a borderless interaction that can range from a significant covalent character (SS interaction) to a mild dipolar interaction (CS interaction) [22,23,24]. In this sense, hydrogen-, and by extension halogen-, bonded systems display interactions that can be thought in-between primary covalent interactions and mere van der Waals contacts [22,23,24,50]. This notion comes out in favor of ERMBs and EDMBs descriptions alike since the H atom has been found to form part of both EDMBs (in boranes) and ERMBs (in most of the strong hydrogen bonds in the [FHF]^−^ or HF_2_^−^ anion [22,23,24]). Therefore, EDMBs and ERMBs are found in electron-precise elements, like H. Third, EDMBs have been suggested to occur in several electron-precise and electron-rich elements. In this context, Vegas and coworkers have already suggested that EDMBs should be present in the linear Si–C–Si bond of the carbocation [Si_2_(CH_3_)_7_]^+^, in the linear Si–O–Si bonds of hexamethyldisiloxane, (H_3_C)_3_–Si–O–Si–(CH_3_)_3_, as well as in the linear Si–O–Si bonds within the [O_3_Si–O–SiO_3_]^6−^ polyanion in solid Sc_2_Si_2_O_7_ silicate [51,52].

A last comment should be made about the different names that have been proposed for two-center bonds with less than two shared electrons, i.e., electron-deficient bonds if we compare them with the electron-precise covalent 2c-2e bonds. This electron deficiency is typically assumed to be also the case for the two-center bonds constituting three-center ERMBs and EDMBs, and it has been used to give account for the half-bond nature of the two-center bonds in ERMBs and EDMBs [1]. In this context, electron-deficient bonds (without any mention of a possible multicenter character) have been proposed to define the large C–C bonds present in carboranes and hydrocarbons [53,54]; in large O–O bonds in H_2_O_2_; and in weak F–F bonds in F_2_. Curiously, the interactions in F_2_ have also been named as protocovalent bonds [55], electron-deficient covalent bonds [56], and CSBs [25,57,58]. More recently, Wuttig and coworkers have claimed that two-center-one-electron (2c-1e) bonds, named by them as metavalent bonds, exist in the crystalline phases of PCMs (SnTe, PbTe), and even in lead halide perovskites [20,59,60,61,62]. This interpretation has been questioned by other authors, who defend that PCMs, mainly chalcogenides composed of valence electron-rich elements, exhibit hyperbonds [63,64,65,66,67,68,69], as previously suggested in other solids made of electron-rich elements, e.g., sc-Sb and TlTe [38]. This situation has led to the current controversy regarding which one of the two models, the metavalent bonding model or the hypervalent (ERMB) bonding model, explains the geometries, bonding features, and properties of the crystalline phases of PCMs. We notice that this divergence has been solved by the EDMB model arising from the UTMB [4,5,9,10].

In summary, the story outlined in the preceding paragraphs suggests that the understanding of the two types of multicenter bonds was far from being complete before the UTMB, and that it is necessary to deeply understand the unconventional multicenter bonds, especially with reference to solids, to resolve the ongoing controversies. This is one of the aims of the UTMB. Moreover, from a practical point of view, there is currently an inflation of chemical terms referring to the same kind of chemical bonding. Trying to pragmatically simplify this mesh, which is especially cumbersome for physicists trying to understand chemical bonding, is another of the aims of the UTMB. In this context, it is interesting to notice that a strong relationship between the ERMB present in hypercoordinated molecules, such as XeF_2_, and the CSB present in many molecules, such as F_2_, has been pointed out [58,70,71], thus supporting the existence of ERMBs as a distinct chemical bond. We want to advance that, in the last section of this manuscript, we will show that CSBs and ERMBs share the same type of bonding and are located in the same region of the ES vs. ET map (the orange region in Figure 2). The main difference is that CSBs are two-center bonds, while ERMBs are three-center bonds. Thus, unlike in ERMBs, no multicenter character (due to electron delocalization between two bonds) is present in CSBs.

## 3. Origins of the UTMB

The UTMB is a daughter of high-pressure studies in material science. Pressure is a highly versatile tool that allows matter compression, leading to an increase in mass density. The most important feature of pressure is that it allows us to finely tune interatomic distances, resulting in an increase in electronic density through an increase in the overlap of electronic clouds. In turn, this forces electron transfers or electron redistributions, e.g., among neighboring atoms, which may lead to changes in interatomic chemical bonding. For instance, it is known within the high-pressure community that most materials, even the elusive elemental hydrogen (H_2_ at room pressure), tend to metallize under compression [72,73]. In other words, it can be considered that materials tend to change their covalent, ionic, or polar covalent bonding at low pressures towards metallic bonding at high pressures. In this compression process, it has been proposed that the appearance of multicenter interactions could be favored [74]. Moreover, even systems with a high electronic density, like metals, suffer changes in chemical bonding under compression. In particular, experiments and calculations have shown that at extremely high pressure (up to 1 Mbar) valence electrons of metals can separate from the core and form pairs of electrons located at the voids of the net formed by the cores, forming electrides [75,76,77]. Therefore, pressure is an inestimable tool to understand changes in chemical bonding, and some of us have made use of this advantage to understand the formation of multicenter bonds by paying special attention to the behavior of secondary bonds (or interactions) [4]; a subject that, in our opinion, has not been properly addressed in previous studies of materials under compression despite multicenter bonds are prone to occur in molecular solids under compression and the effect of pressure in those solids, particularly pressure-induced polymerization, has been studied for decades.

Interestingly, pressure is a tool that allows us to unify interactions in molecules and solids; i.e., it promotes a unified view of chemical bonding in organic chemistry, inorganic chemistry, supramolecular chemistry, organometallic chemistry, and solid-state chemistry, since pressure promotes, in general, the decrease in interatomic distances in any material. Notice that the trans influence or trans effect (two chemical terminologies) that occurs in D-A interactions in supramolecular chemistry, leading to the alteration (usually weakening) of the primary (intramolecular) chemical bonds inside the donor and the acceptor, when the intermolecular interactions become stronger, is analogous to the trans influence that occurs in solid-state chemistry, in which secondary bonds can promote changes in the primary bonds of solids, as demonstrated in the UTMB [4,5].

The way in which changes in chemical bonding proceed under compression has been studied in many theoretical works. It has been suggested that the transformation of a secondary non-covalent interaction (typical intermolecular CS interaction) into an SS interaction can be accurately assessed by the determination of the number of electrons shared between two atoms in that bond (a number equivalent to twice the delocalization index) [78]. This quantification is easily obtained from DFT simulations, although its experimental determination is not straightforward. In this context, it must be mentioned that the experimental study of the pressure-induced change in chemical bonding mainly comes from resistivity changes: a switch from insulating to metallic conduction, and from a negative to a positive temperature coefficient of resistivity, is assumed to be a confirmation that covalent or polar-covalent bonds (semiconducting behavior) transform into metallic ones (metallic behavior). Therefore, more specific experimental methods and investigations are needed to determine the number of shared electrons between two atoms in materials to experimentally quantify the change in chemical bonding with pressure.

It is important to notice the extreme efficiency of pressure in increasing the electronic density, as it allows a much larger increase in electronic density than any of the other methodologies, namely temperature, reduction, or chemical substitution (note that electronic density increases when an atom is replaced by a heavier isoelectronic analog within a group of the periodic table, e.g., substitution of As by Sb or by Bi). Furthermore, high-pressure studies are in a privileged position to study changes of chemical bonding in materials, as they allow us to observe these changes in small steps, like individual photograms in a movie, where each step is a given pressure point. For all the above reasons, high-pressure techniques have allowed us to tackle the problem of understanding the changes of chemical bonding in materials upon compression, which has finally led to the development of the UTMB [4,5].

It is necessary to comment that the UTMB originated in our attempt to solve the controversies regarding the chemical bonding of some compounds at given pressures. In particular, we were interested in knowing whether the bonds in the crystalline phases of PCMs (the rocksalt (rs) phase of SnTe, PbS, PbSe, and PbTe) could be explained either by the metavalent or the hypervalent bonding models. To answer this question, some of us carried out DFT calculations in their simplest analogs, i.e., in elemental pnictogens (also in chalcogens) [4]. These DFT simulations were complemented by a thorough study of the topology of the electron density by means of Bader’s quantum theory of atoms in molecules (QTAIM) [79,80]. These initial works have recently been extended to studies of the binary A^IV^X^VI^ and A_2_^III^X_3_^VI^ compounds related to PCMs [10], in which additional methodologies, such as the calculation of the energy densities at the bond critical point, have been shown to provide additional information for the identification of the EDMBs present in the highly coordinated phases of the above-mentioned elements and binary compounds. In this context, we want to stress that the A^IV^X^VI^ binary compounds are isoelectronic to group 15 elements (pnictogens), and it is well known that elemental phosphorus and arsenic undergo a phase transition to the sc phase upon increase of pressure [81,82,83]. Therefore, we considered that the bonding in the sc phase of pnictogens should be the same as that of isoelectronic PCMs, since all atoms in both sc and rs phases show an octahedral coordination. This high atomic coordination (assumed to violate the 8–N rule if all bonds were covalent bonds) was related to the extraordinary properties of PCMs [20,59,60,61]. In this regard, the behavior of pnictogens (also of chalcogens) under compression has been crucial to understand the nature of the exotic bonding found in their pressure-induced highly coordinated phases and hence to understand the process of formation of the unconventional bonds (EDMBs) present in the highly coordinated crystalline phases of PCMs at room pressure.

Several facts observed at high pressure led us to think that the current metavalent and hypervalent bonding models were not adequate to account for the chemical bonding present in the crystalline phases of chalcogenide-based PCMs. First, binary compounds that were not PCMs at room pressure, like rhombohedral GeTe, and other related materials, such as rhombohedral As isoelectronic to GeTe, exhibit “soft” high-frequency vibrational modes, i.e., high-frequency vibrational modes with negative pressure coefficients. This feature was known to occur in several pnictogens (As and Sb) and chalcogens (Se and Te) from the early Raman scattering studies under compression in the late 1960s and 1970s [84]. Since the high-frequency vibrational modes in common covalent bonds of semiconductors (Si, Ge, GaAs, etc.) usually increase in frequency (or “harden”) under compression, the “softening” of the high-frequency vibrational modes in pnictogens and chalcogens was considered an anomalous behavior, which was ascribed in those days to the weakening of the covalent bond in these elements under compression. This weakening was attributed to a possible loss of electron charge with increasing pressure, but the final destination of the lost electron charge (present in the uncompressed covalent bonds) was not explained. Second, another anomalous behavior was observed under compression in certain elements, such as Se. The bond distance of the covalent Se–Se bond in trigonal Se increases with increasing pressure [85]. This is just the opposite behavior of common covalent bonds in semiconductors (Si, Ge, GaAs, etc.), whose bond distances decrease under compression. Thus, the trend in trigonal Se was an anomalous behavior for which no explanation was offered. It was just considered to be aligned with the anomalous expansion of the *c* axis of the hexagonal unit cell in trigonal Se (at the expense of the contraction of the *a* and *b* axes). The only explanation offered for this phenomenon was that pressure induced a strong compression of secondary bond distances (mainly along *a* and *b* axes) at the expense of a slight increase in length of the *c* axis, which is related to the slight increase in the primary covalent Se–Se bond length. This behavior was consistent with the weakening of the covalent bond, as Raman measurements already suggested. However, the relationship between the increase in length and the loss of electronic charge of the Se–Se covalent bond under compression remained unexplained, since it was not detailed where the lost electronic charge by the covalent bond was transferred.

The transference of the electronic charge lost by the covalent bonds of pnictogens and chalcogens under compression was addressed in the study of dense pnictogens and chalcogens, whose aim was to solve the issue of the chemical bonds in the crystalline phases of PCMs [4], and which led to the development of the UTMB [5]. In that work, the methodologies of both the proponents of the metavalent bonding model (who used density-based methods) and of the hypervalent bonding model (who used orbital-based methods) were evaluated. With this open approach, the relationship between the anomalous soft high-frequency vibrational modes, the increase in the bond length, and the loss of electronic charge in the covalent bond was explained. The explanation is simple: the primary short covalent bonds in the low-coordinated phases of pnictogens and chalcogens weaken under compression because part of the electronic charge of the covalent bond is transferred to the secondary long non-covalent bonds. **Now we know the final destination of the electronic charge lost by the covalent bond!** The loss of the electronic charge (and consequently of the strength) of the primary bond is related to its increase in length in the same way as the gain of the electronic charge (and consequently of the strength) of the secondary bond is related to its decrease in length. The charge transfer between bonds is related to the trans influence between the two bonds. In other words, the transfer of charge between the primary and secondary bonds is part of a multicenter interaction between two correlated (almost collinear) bonds. This multicenter interaction present in the low-coordinated phases of pnictogens and chalcogens ultimately can lead to the formation of EDMBs in the highly coordinated phases (ideally when both primary and secondary bonds equalize in length and electronic charge) [4]. A more detailed explanation is given below in relation to the different stages of the formation of the EDMBs. 

Since the same process explained in the above paragraph occurs for PCMs [10], in the end it was shown [4,10] that the low-coordinated phases of pnictogens and chalcogens, as well as of the crystalline phases of A^IV^X^VI^ and A_2_^V^X_3_^VI^ binary compounds that are not PCMs at room conditions (but become PCMs at high pressure), show multicenter interactions that develop into EDMBs when highly coordinated phases are reached upon compression. In other words, it was found that previous metavalent and hypervalent models were right and wrong at the same time, as thoroughly discussed in a comprehensive work [9]. In that work, it was commented that the proponents of the metavalent bonding model for PCMs were right in signaling the electron-deficient character of the interaction, but underestimated the multicenter nature of the bonding. This seems to have been reconsidered in a recent work in which the authors have not mentioned the word “metavalent bonding” in PCMs and catalog them only as 2c-1e bonds [7]. On the other hand, in ref. [9] it was commented that the proponents of the hypervalent bonding model for PCMs were right in highlighting the multicenter nature of the interaction, but erroneously considered that the interaction was electron-rich and not electron-deficient in character. Three main features led the proponents of the hypervalent bonding model to consider them as ERMBs and not as EDMBs: (i) the fact that PCMs are made of electron-rich elements [1,38], (ii) the linear bonds present in PCMs (similar to those present in ERMBs, see Figure 1a), and (iii) the negative value of the integrated crystal orbital bond index for three centers, ICOBI(3c). These features were discussed in our works [4,5,9,10] and later briefly commented. All in all, a third way was proposed by some of us: bonds in the crystalline phases of PCMs are EDMBs (neither ERMBs nor metavalent bonds) [4,5,9,10]. The EDMBs present in the highly coordinated crystalline phases of PCMs and related materials can explain at once the geometries, bonding features, and the properties of these compounds, thus proposing a common framework to understand bonding in these technologically relevant materials.

As already mentioned, the work on pnictogens and chalcogens under compression [4] demonstrated that EDMBs can be found in electron-rich elements, contrary to what was traditionally assumed [1,38]. Our results confirmed the goodness of the electron-counting rule of Papoian and Hoffmann, which dictates the conditions that must be fulfilled to form multicenter bonds [38], and at the same time demonstrated that their interpretation of chemical bonds in some solids with valence electron-rich elements, such as sc-Sb, Li_2_Sb, BaZnSb_2_, and TlTe, as governed by ERMBs [38], was not correct because these solids feature EDMBs (notice that both Te–Te–Te ERMBs (3c-4e bonds) forming trimers and –Te–Te–Te–Te– EDMBs (2c-1e bonds) forming in infinite linear Te chains are present in TlTe) [5]. Moreover, it was shown that elemental polonium at room conditions is a paradigmatic example of an elemental solid with only EDMBs [4], since EDMBs are also present in elemental boron at room conditions, but in coexistence with covalent bonds [86].

It must be stressed that the multicenter character of the bonding present in the sixfold-coordinated solid phases of pnictogens and chalcogens was verified by the evolution of the bonding descriptors in these solids under compression without the calculation of any multicenter bond index [4]. Some researchers surely will not consider the presence of multicenter bonds unless a multicenter bond index can verify it. However, we must note that multicenter bond indices have to be validated before their widespread use by the scientific community. How are we going to validate these multicenter bond indexes if there are no standards for validation? In this context, we have followed a different approach thanks to high-pressure techniques since the appearance of a pressure-induced multicenter interaction, followed by the development of a multicenter bond, already provides the certification that we are facing the mechanism of formation of a multicenter bond [5,6]. This certification is independent and prior to the validation of any multicenter bond index. In fact, our examples of pressure-induced multicenter bonds are going to be the testbed examples with which those multicenter bond indexes can be validated in future. In other words, the multicenter character of the bonding in those highly coordinated phases is verified by the previous existence of a multicenter interaction (related to the trans influence) between the primary and secondary bonds present in the twofold- and threefold-coordinated (low-coordinated) phases of chalcogens and pnictogens, respectively. In this respect, several researchers have asked us to confirm the multicenter character of the bonds using a multicenter bond index for solids, similar to those known for molecules. It must be stressed that at the time of the publication of the UTMB, there was no multicenter bond index available for solids that could be obtained from density-based calculations. A new multicenter bond index for solids obtained from density-based calculations, such as CRITIC2 [87] seems to have been recently developed, but it is still not widely available for researchers to our knowledge [7]. On the other hand, a multicenter bond index for solids, such as the ICOBI(3c), as obtained from the LOBSTER code [88,89], can be obtained from orbital-based calculations [65,68,69]. In particular, the multicenter character of the bonding in the highly coordinated phases of pnictogens and chalcogens was confirmed by the calculation of this index [4]. The ICOBI(3c) value was found on the order of −0.1 [4], a value which is of the same order as that found in PCMs, e.g., rs-GeTe and rs-PbTe [68,69]. According to Dronskowski and coworkers, the negative ICOBI(3c) value indicates the electron-rich nature of the multicenter bond in the above *solids*, because this value is negative (positive) in well-known electron-rich (electron-deficient) *molecules* [65]. The main difference between the ICOBI(3c) values in well-known ERMBs, such as those present in XeF_2_ and I_3_^−^, and in solid pnictogens, chalcogens, and PCMs, is that in the first two systems the values are around −0.35, while in the mentioned solids they are around −0.1. On the other hand, the electron-deficient character of the bonding in the highly coordinated phases of pnictogens, chalcogens, and PCMs was suggested by the calculated number of electrons shared (ES) between two atoms, which is typically around 1 [4,10,20], i.e., half of that expected for a 2c-2e bond and also a value that is too low for an ERMB, e.g., XeF_2_ [20,62].

**Figure 4:**
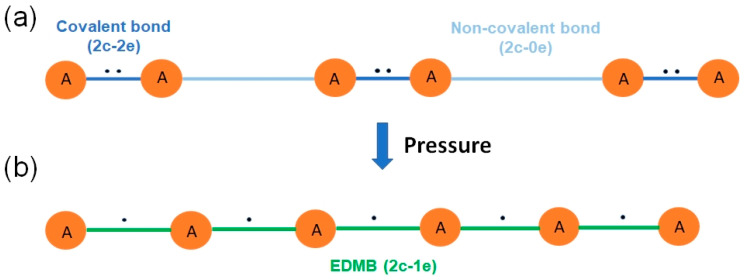
(**a**) Schematic representation of a linear chain of A atoms showing short, strong A–A bonds and long, weak A···A bonds. Atoms, bonds, and electrons are represented as filled circles, solid bars, and black dots, respectively. In this diatomic chain, it can be assumed approximately that the short A–A bonds are 2c-2e bonds while the long non-covalent A···A bonds are 2c-0e bonds; i.e., there is no electron shared between the two atoms as it would occur in a closed-shell interaction. (**b**) The same chain compressed (~A~A~A~) shows all bonds of equal length and strength as a result of a pressure-induced polymerization process. In this atomic chain, 2c-1e EDMBs result as a redistribution of the electrons in the diatomic chain since all bonds are equal.

Due to the contradictory conclusions provided by the two above-mentioned chemical bonding descriptors in the highly coordinated crystalline phases of pnictogens, chalcogens, and PCMs, with ICOBI(3c) suggesting ERMBs and ES suggesting EDMBs, the pressure dependence of the primary and secondary bonds in the low-coordinated phases of pnictogens and chalcogens [4] was thoroughly studied. The results concluded that:

(1) The highly coordinated phases of pnictogens and chalcogens feature multicenter bonds. As already mentioned, the multicenter character of the bonds in these phases was explained by the mechanism of bond formation in the low-coordinated phases (without attending to the calculated multicenter bond index). In particular, the multicenter bonds (in the highly coordinated phases) derive from the equalization of primary and secondary bonds (in the low-coordinated phases) as a consequence of the trans influence between the secondary non-covalent bonds and the primary covalent bonds. This means that the secondary bonds affect the primary bonds in those low-coordinated phases. More specifically, it is found that, upon compression, the primary covalent bonds weaken and increase in length at the same time as the secondary bonds strengthen and decrease in length when the multicenter interaction or trans influence takes place. Ultimately, the multicenter interaction leads to the formation of a single type of multicenter bond in the highly coordinated phases of pnictogens and chalcogens, as proven by the equalization of the parameters characterizing the primary and secondary bonds (in the low-coordinated phases) with increasing pressure.
(2) The bonding in the highly coordinated phases of pnictogens and chalcogens has an electron-deficient (3c-2e or 2c-1e) character. This character was demonstrated by the mechanism of bond formation in the highly coordinated phases, showing the charge transfer from the primary covalent bond to the secondary non-covalent bond. Once the primary covalent and secondary non-covalent bonds equalize, a three-center bond is formed (in the highly coordinated phases). This new three-center bond is formed by the two electrons already present in the primary covalent bond in the low-coordinated phases. The progressive charge transfer of one electron from the primary covalent bond to the secondary non-covalent bond (in the low-coordinated phases) is observed when ES values of both primary and secondary bonds are plotted. In the end, the two electrons previously belonging to the primary bond are now shared by two equal and collinear 2c-1e bonds, forming part of a 3c-2e bond (in the highly coordinated phases). In other words, the new bond in the highly coordinated phases (formed by the contraction of the secondary bond in the low coordinated phases) occurs due to the donation of one of the two electrons present in the opposite (in linear or quasi-linear configuration) covalent 2c-2e bond of the low coordinated phase [4]. Another way of explaining this bond formation process is that two linked 2c-1e bonds (forming a 3c-2e bond) are formed from two original collinear bonds upon compression (see Figure 4): a 2c-2e bond (the strong primary covalent bond) and a 2c-0e bond (the weak secondary non-covalent interaction). Unlike the charge reorganization leading to the formation of ERMBs [37,39,40], which has been already reviewed [40,63,64,65], to the best of our knowledge, the charge transfer process leading to the formation of EDMBs has not been previously reported. Interestingly, the same kind of bonds (EDMBs) found in sc-As (above 25 GPa) were also found in sc-Po (α-Po) at room pressure. Moreover, a similar charge transfer process (leading to the formation of EDMBs) was found to occur in pnictogens and chalcogens under compression [4]; however, chalcogens were found to tend to the rhombohedral (rh) phase of Po (β-Po) at high pressures and not to the sc phase, as previously assumed.

These two conclusions together led us to propose the existence of EDMBs in the highly coordinated phases of pnictogens and chalcogens. Once these consistent results on pnictogens and chalcogens under compression were obtained [4], the existence of EDMBs in the related highly coordinated rs phases of PCMs has also been proved [10].

Now let us address the issue with the ICOBI(3c) values since we have commented that ES and ICOBI(3c) values suggested a different nature of the studied bonds in the highly coordinated phases of pnictogens, chalcogens, and PCMs. As already mentioned, the ICOBI(3c) values in the highly coordinated phases of pnictogens and chalcogens are ca. −0.1, i.e., of the same order as that found in PCMs, e.g., rs-GeTe and rs-PbTe [68,69]. Therefore, a reasonable doubt was cast in ref. [4] regarding the interpretation provided by Dronskowski and coworkers of the −0.1 value in PCMs. This doubt has been clarified by a recent study of PCMs [7], a recent work of AX_3_ halides under compression, in particular of CsI_3_ [17], and a very recent manuscript regarding metallic bonds in main-group elements [19]. It is worth mentioning what has been found in these three works.

CsI_3_ is a molecular solid that shows isolated asymmetric I_3_^−^ anions in the orthorhombic *Pnma* phase at room pressure and is characterized by ionic bonds between Cs^+^ atoms and I_3_^−^ anions and by an I–I–I ERMB in the linear I_3_^−^ anions (Figure 1a) [65]. Interestingly, CsI_3_ has been experimentally found to undergo several pressure-induced phase transitions, first to a hexagonal *P*-3*c*1 phase, characterized by interacting symmetric I_3_^−^ anions (aligned along the *a*-, *b*-, and *c*-axes), and then to a cubic *Pm*-3*n* phase, characterized by infinite linear iodine chains, I_∞_, along the three axes, generated by the polymerization of the I_3_^−^ units of the *P*-3*c*1 phase, in which all I–I bonds show the same bond length [90,91,92]. In ref. [17], it has been shown how the ERMBs of the isolated asymmetric I_3_^−^ units of the *Pnma* phase transform into 2c-1e EDMBs in I_∞_ (they can also be seen as concatenated 3c-2e bonds along the infinite chain, see Figure 1c). In other words, the infinite linear iodine chain is fundamentally characterized by the sharing of one electron between two iodine atoms. In this way, it was shown how pressure can lead to a change from ERMBs to EDMBs in CsI_3_ (also in other AX_3_ halides), consistent with the pressure-induced change in the properties of this compound. Interestingly, during the formation of the linear chains, the ICOBI(3c) value changes from −0.4 (when ERMBs are present in I_3_^−^ units) to −0.1 (when EDMBs are present in the I_∞_ chain); a result that suggests that low negative values of the ICOBI(3c) can be associated with EDMBs instead of ERMBs. Likewise, Gatty and Raty have shown a potential issue with the calculation of ICOBI(3c) with the LOBSTER software (version 5.1.0) that can lead to negative values for some EDMBs [7]; an issue that has been denied by Dronskowski and coworkers [93].

Regarding the value of ICOBI(3c), it must be finally stressed that a recent work has evaluated the possibility that some metallic bonds may exhibit a multicenter character along certain interatomic directions [19]. It has been verified that the value of ICOBI(3c) of several elements with metallic bonds exhibits a small negative value (typically between 0 and −0.1). Since it is well known that metallic bonds are electron deficient in character, these results definitively prove that electron-deficient bonds, either in EDMBs or metallic bonds, yield small negative values of ICOBI(3c). Therefore, the interpretation of the chemical bonds in PCMs as ERMBs [68,69] based on the negative value of ICOBI(3c) seems to be totally unfounded.

In summary, the works about the effects of pressure on pnictogens and chalcogens [4], A^IV^X^VI^ and A_2_^V^X_3_^VI^ binary compounds [10], CsI_3_ [17], and metal iodates [12,13,15], as well as other examples in ref. [5], have shown, against previous assumptions [1,38], that EDMBs are possible in electron-rich elements (pnictogens, chalcogens, and halogens) forming infinite-linear atomic chains in one, two, or three dimensions.

These results in solids were certainly interesting and led us to question if linear ERMBs could be found in extended solids. In this context, ERMBs are known to occur in molecules formed by electron-rich elements, and most (if not all) ERMBs are found in linear or quasi-linear trimers as 3c-4e bonds (Figure 1a) and not in larger linear molecules [40]. In this context, some of us realized that one clear example of the impossibility of linear ERMBs in systems larger than three atoms was provided by the well-known family of polyiodides. These molecules (I_3_^−^, I_5_^−^, I_7_^−^, etc.) are known to have complex geometries [94]. Linear or quasi-linear arrays of I atoms are usually observed for I_3_^−^, rarely observed for I_5_^−^, and totally absent for larger linear molecules. In fact, I_5_^−^ is known for typical angular V- or L-shape geometry and I_7_^−^ for its pyramidal or Z-like geometry. All these geometries were nicely explained by considering the role of EDMBs and ERMBs; in particular, the impossibility of forming ERMBs larger than three centers [16].

The absence of ERMBs in linear molecules larger than three centers led us to think that this situation was related to the violation of the octet rule, which likely imposes some energy and symmetry restrictions, as those found for polyiodides (also to be applied to polyhalides) [16]. This issue was commented on in the work presenting the UTMB that addresses the origin and mechanisms of formation of ERMBs and EDMBs [5]. In that work, it was shown that the multicenter bonds have the same origin, i.e., the merging of two two-center bonds, a primary strong bond (typically an SS interaction as a polar covalent bond) and a secondary weak bond (typically a CS interaction as a non-covalent bond), into a single three-center bond, which can be eventually extended to more centers. As paradigmatic examples of simple multicenter bonds, the formation of EDMBs in diborane (B_2_H_6_) molecule—such as the link of two borane (BH_3_) molecules—as well as the formation of ERMBs in the I_3_^−^ anion—such as the link of an I_2_ molecule and an I^−^ ion—were dissected in ref. [5].

The UTMB clarifies that the formation of EDMBs and ERMBs proceeds in different ways [5]. As commented previously, while the formation of ERMBs agrees with the common assumption that the two non-bonding electrons of a single lone electron pair (LEP) become bonding electrons that help to establish the 3c-4e bond [37,39,40,63,64,65], the formation of EDMBs involves the donation of one electron of the primary 2c-2e bond to the secondary bond, so that both bonds become 2c-1e bonds; i.e., they form part of a joint 3c-2e bond [4]. To the best of our knowledge, this mechanism was not previously suggested, as already commented. Moreover, if there are non-bonding electrons forming part of the LEP that participates in the original secondary bond, the formation of EDMBs requires, in general, that these electrons remain as non-bonding electrons in the new three-center bond due to the delocalization of the LEP, e.g., as it occurs in sc-As and in α- and β-Po [4]. Recently, the LEP delocalization has been commented to occur in other systems [95]. In this context, we want to make clear an issue regarding the formation of EDMBs in the highly coordinated phases of pnictogens and chalcogens. According to the electron-counting rule established by Papoian and Hoffmann for the formation of multicenter bonds [38], the formation of EDMBs in sc-As and sc-Sb (in ref. [38] it was assumed that they were ERMBs) needs five valence electrons, as indeed occurs in pnictogens. The EDMBs are formed with the three *p*-type electrons, while the *s*-type electrons do not participate in the bonding and remain as non-bonding electrons [38]. In this context, the process of formation of six equal bonds in sixfold-coordinated sc-As from the original threefold-coordinated rh-As requires that the three short covalent bonds in rh-As donate one of the two electrons to the three long non-covalent bonds in rh-As, thus resulting in six equal As–As bonds with only one electron between two atoms in sc-As. This means that the LEP of As in rh-As plays no role in the formation of the EDMBs in sc-As. In fact, the electron localization function (ELF) picture shows that the LEP becomes delocalized with increasing pressure in rh-As, and the two *s*-type electrons of the LEP do not participate in bonding, despite the LEP being redistributed into six ELF lobes along the new bonds in sc-As [4]. Now we will comment on the process of formation of six equal bonds in sixfold-coordinated rh-Te from the original twofold-coordinated hexagonal (h) Te. Although the case of Te bears similarities with the mechanism described for As, some differences arise due to Te being a chalcogen and thus having six valence electrons (instead of five). This means that the sc phase with six equal perpendicular bonds is a less favorable configuration according to the electron-counting rule [38], because an extra *p*-type electron is present. The formation of six equal bonds in the rh-Te can be described as follows. First, it is important to note that each Te atom in h-Te has two bonding electrons (one for each of the two covalent bonds) and four non-bonding electrons (in two LEPs that are almost aligned with two of the four secondary non-covalent bonds). Thus, the two short covalent bonds in h-Te donate one of the two electrons to two of the four long secondary bonds during the mechanism of EDMB formation. Clearly, this electron transfer cannot result in the formation of six equal 2c-1e Te–Te bonds in rh-Te since one more electron is necessary to satisfy the three shared electrons, as in As (if we consider that the *s*-type and *p*-type electrons of the two LEPs in h-Te remain as non-bonding electrons and the LEP becomes delocalized, as we have already described for *s*-type electrons in the only LEP of As). This reasoning indicates that the formation of six equal EDMBs in rh-Te can only take place if one of the two LEPs of h-Te (with *p*-type electrons) donates one of its two non-bonding electrons to the two remaining secondary bonds in h-Te that do not receive charge from the covalent bonds. In other words, the formation of EDMBs in rh-Te needs the contribution of one *p*-type electron originally owned by one of the two LEPs present in the Te atoms of h-Te. The remaining *p*-type electron from the donating LEP and the two *s*-type electrons of the other LEP in the Te atoms of h-Te become delocalized and redistributed and, thus, do not participate in the bonding (as in sc-As). This is verified by the ELF, which shows how these three electrons (two of the LEP and the extra *p*-type electron) are distributed in a toroidal basin in rh-Te [4].

In summary, the UTMB emerged from investigations into the effect of pressure in various solids, revealing that EDMBs, as 2c-1e bonds forming part of concatenated 3c-2e bonds in infinite linear atomic chains, occur in solids with hypercoordinated atoms of valence electron-rich elements. The discovery of EDMBs in these systems led us to question the nature of ERMBs in solids. We concluded that linear ERMBs can only be found as linear (or quasi-linear) 3c-4e bonds. Further investigation led to the clarification of the common origin and different formation mechanisms of EDMBs and ERMBs, as exposed in the UTMB [5].

## 4. Multicenter Bonds and Pressure: A Qualitative View

The formation of EDMBs, as exposed in the UTMB, can be grasped, up to a certain extent, using the simplified example of the infinite linear chain of generic A atoms with alternating strong (primary) A–A and weak (secondary) A···A interactions (see Figure 4a). Even though the following representation is not a formal approach, we believe that it can be helpful as a visualization and conceptualization tool. Notation: – and ··· denote primary- and secondary-bond types. In this context, it must be noticed that analogous classic 1D models are used in solid-state physics for deriving the crystal lattice mode vibrations in the harmonic approximation [96].

Let us imagine the mentioned infinite linear chain of generic A atoms with alternating strong and weak interactions as a set of tight and loose springs, in which there is an inverse relationship between the length and the strength. In these conditions, the expected effect of a compression exerted along the chain would then be an overall contraction of the chain, with loose springs being more compressed than tight ones. Eventually, an infinite linear atomic chain ~A~A~A~, being ~ EDMBs, would be formed (Figure 4b), in which all atoms are bonded at left and right sides with the same bonds, i.e., the same length and strength.

One of the reasons why atomic chains deviate from the ~A~A~A~ symmetric behavior, giving instead rise to more and more aliasing of the long and short interactions, is to be found in the Pauli repulsion. At a certain point, the tight springs experience extremely strong repulsion, eventually to the point of springing back and elongating at the expense of the loose ones. In physical terms, we consider this behavior as a Peierls distortion of the more symmetric geometry of the infinite linear atomic chain with equal bond distances and equal interatomic forces.

Another possible argument is the visualization of bonds via the notion of electronegativity equalization [97]. The bonding electrons constitute the above tight springs, while LEPs involved in the weak interactions constitute the loose ones. Upon compression, the compressed primary bonds find themselves with excess electronic density (with respect to the new interatomic distances), while the compressed secondary bonds find themselves lacking electronic density (with respect to the now shorter interatomic distances). A “spill” of the excess electronic density from one side to the other is likely expected. As the short bond elongates (decreases in bond order), the long one shortens (increases in bond order). The overall bonding picture is less and less concerned with the original primary short A–A bond and the secondary long A···A interaction and becomes growingly aware of the ~A~A~A~ multicenter picture. Likewise, the fact that electronegativity must change with pressure can be envisaged [98]. The fact that pressure, by increasing overall orbital overlap, fosters multicenter bonds is also reasonably intuitive from these simple pictures.

In the framework of the UTMB, the above points are formally described in the different stages of formation of a multicenter bond due to the increase in electronic density (e.g., by applying pressure).

At stage 1 (at the beginning, when there is a small electronic density), strong and weak interactions are so different that they behave independently as a set of springs with very different force constants, so the loose springs (secondary bond) contract more than the tight springs (primary bond) under compression. At some value of electronic density (stage 2), if there is a correlation between both interactions (trans effect), the tight spring ceases contracting with pressure and rather starts elongating (“anomalous” behavior), while the loose spring keeps contracting. When this occurs, at some value of electronic density, the two bond distances become equal in length and strength, i.e., EDMBs (stage 3). From that point onwards, the response of the system to pressure returns “normal” as there are no more adjustments to the subtending chemical bonds to be made as they already converged to the same bonding type (the EDMB). Therefore, once formed, EDMBs contract in a normal way with increasing pressure.

The UTMB [5] details these aspects as well as the distinction between ERMBs and EDMBs, overall providing a more coherent picture of the whole of chemical bonding. A more formal introduction to UTMB is given below.

To conclude this section, we want to remark that the disappearance of the Peierls distortion in an infinite linear atomic chain with increasing pressure, as already proposed by Burdett [99], has been nicely exemplified by recent studies in dense AX_3_ halides that show the pressure-induced formation of infinite linear halogen chains, X_∞_, when aligned X_3_^−^ units are compressed and the cubic *Pm*-3*n* phase is reached [90,91,100,101,102]. In particular, the polymerization of the I_3_^−^ units in CsI_3_ has been shown to lead to a change from ERMBs (present in the I_3_^−^ units at low pressures) towards EDMBs (present in the I_∞_ chain at high pressures) [17].

## 5. Introduction and Extension of the UTMB: The Inclusion of the Charge-Shift Bonding

In Section 1 and Section 2, we have commented on the conventional covalent, ionic, and metallic bonds and also on the two types of multicenter bonds known since 1950, especially the three-center bonds. Since multicenter bonds extend to more than two atoms, it is natural to think that three-center bonds, the smallest possible multicenter bond, can be formed from (or thought as) the combination of a two-center bond plus a delocalized interaction. This subject has been addressed in Section 2 and Section 3, where we have commented that the formation of EDMBs and ERMBs, in particular, the three-center bonds, was illustrated in ref. [5] using the examples of the formation of EDMBs in diborane (B_2_H_6_) molecule, as well as the formation of ERMBs in the I_3_^−^ anion. In addition, the formation of EDMBs has been explained in pnictogens and chalcogens according to ref. [4] (later extended to PCMs [10]). Furthermore, Section 3 and Section 4 have highlighted the importance of pressure in elucidating the multicenter nature of bonding in many materials.

In this section, we aim first to provide a brief introduction of the principles leading to the UTMB. This is done by resorting to common concepts and rules in chemistry. Later, we will extend the UTMB to include not only EDMBs and ERMBs together with covalent, ionic, metallic, and resonant bonds, but also to include the CSBs, emphasizing their strong relationship with ERMBs.

It is well known that boron and boron hydrides typically feature EDMBs giving rise to highly coordinated atoms, and that XeF_2_ and I_3_^−^ molecules feature ERMBs, also showing atoms with a coordination higher than expected. Therefore, the first approach to multicenter bonding is to wonder which kind of chemical bonding occurs in systems with hypercoordinated atoms (hypercoordination understood as a violation of the 2–N or 8–N rules for covalent bonding). The first answer to this question is simple: systems with hypercoordinated atoms cannot be described only by classical covalent or polar covalent bonds and, provided that they are not ionic bonds, most likely exhibit multicenter bonds.

In this context, two keys are fundamental for understanding the formation of multicenter bonds. The first key is “the electron delocalization” since multicenter bonds cannot occur in systems with only localized electrons between two atoms, as it occurs in ionic, polar covalent, and covalent bonds. In other words, multicenter bonds are characterized by partially delocalized electrons along certain bonding directions (note in this context that we consider metals to feature a full electron delocalization). In particular, the electron delocalization is restricted to three atoms in ERMBs (3c-4e bonds), like in 1D XeF_2_, HF_2_^−^ and I_3_^−^ and larger polyiodides [5,16,18]. In contrast, the electron delocalization in EDMBs shows two options, one involving delocalization along three atoms (3c-2e bonds) in systems with a close (typically a triangular-like) configuration, such as H_3_^+^ and B_2_H_6_ (Figure 1b) [5], and a second one in which the electron delocalization can be expanded along a line (2c-1e bonds) in systems with an open (quasi-linear- or linear-like) configuration, e.g., along an infinite atomic chain in sc-As, α-Po, and β-Po [4], in the crystalline phases of PCMs, like rs-GeTe and rs-PbTe [14], or in the infinite linear halogen chain, I_∞_, in polyiodides and cubic CsI_3_ (Figure 1c) [16,17,18]. The second key is to understand that electron delocalization can occur in hypercoordinated atoms, i.e., atoms that have more bonds than expected to satisfy the doublet/octet rule with ionocovalent or polar covalent bonds. In this context, the hypercoordination of main-group elements has been related to the violation of the 8–N rule for atomic coordination.

The formation of multicenter bonds in hypercoordinated systems with a deficiency of electrons in bonding was already recognized by W.A. Harrison and coworkers [47]. It is well known that the doublet/octet rule is satisfied with the formation of ionocovalent bonds or polar covalent bonds. In this context, the distribution of the eight electrons in the valence shell must be compatible with the principles of Quantum Mechanics, so the simplest distribution of electrons is to form four electron pairs (two electrons of opposite spin per pair) separated as much as possible according to the VSEPR theory, i.e., distributed along the four diagonals of the cube in the arrangement of tetrahedral *sp*^3^ hybrid orbitals. Note that a modification of this rule to see the four electron pairs as two quartets was proposed by Linnett [103,104]. Therefore, the completion of the octet by the formation of covalent bonds is mainly linked to the formation of *sp*^3^ hybrid orbitals, which in turn is related to an atomic coordination between one (one for hydrogen and halogens, two for chalcogens, and three for pnictogens) and four (for group 14 elements and their binary semiconducting analogs). Consequently, an atomic coordination higher than four, as it happens in the high-pressure phases of compounds that are ionocovalent at room pressure, necessarily leads to the formation of electron-deficient (multicenter) bonds (with less than two shared electrons between two atoms), with a broken *sp*^3^ hybridization of *s* and *p* orbitals [47]. For instance, the breaking of the *sp*^3^ electronic configuration and the formation of a system with *sp*^2^ + *p* electronic configuration, i.e., with trigonal bipyramidal geometry, is one of the possible configurations that can accommodate one linear multicenter bond (formed with the non-hybridized *p* orbital) and either three covalent bonds, three lone electron pairs (LEPs), or a mixture of both covalent bonds and LEPs (in a *sp*^2^ hybrid orbital), in agreement with the VSEPR theory [5,8]. As commented in ref. [5], the trigonal bipyramidal geometry is a typical configuration of an atom with: (i) twofold coordination, such as the central I atom in I_3_^−^ units that forms an ERMB (e.g., in *Pnma*-CsI_3_), any I atom in I_∞_ forming EDMBs (e.g., in cubic CsI_3_), and (ii) fivefold coordination, such as the P atom in the PF_5_ molecule, which forms one axial F–P–F ERMB and three covalent bonds in the plane perpendicular to the ERMB [71]. Another bonding configuration for multicenter bonds corresponds to the formation of a system with *s* + *p*^3^ orbitals, i.e., a system with non-hybridized *s* and *p* orbitals. This is a typical configuration with three perpendicular multicenter bonds (with participation of *p* electrons and not of *s* electrons) and that leads to sixfold atomic coordination, such as Po in α-Po, as As in sc-As, and as both cations and anions in the rs phase of PCMs.

A notable exception to the above *s*-*p* hybridization reasoning is constituted by H, which, when fulfilling the role of the central atom of a multicenter bond (e.g., FHF^−^), displays a classical and linear twofold hypercoordination. In this case, the explanation of linearity cannot be ascribed to *p* orbitals but instead to the breaking of the spherical symmetry of the *s* atomic orbital upon the formation of collinear and opposite σ and σ* molecular orbitals in a σ bond. These aspects constitute basic knowledge for the understanding of strong and linear hydrogen bonds [105].

It must be noted that the formation of multicenter bonds, with the corresponding coordination geometries described in the UTMB [5], is very much related to Gutmann’s rules of D-A interactions in supramolecular chemistry [44,45]. These are a set of empirical rules used to rationalize bond lengths and charge-density variations in molecular D-A complexes. The first rule is a restatement of the trans influence (or trans effect): the stronger the D-A interaction is (in terms of bond length), the more significant is the weakening (elongation) of preexisting bonds in the D and A species. The second rule deals with the changes (lengthening or shortening) brought about in σ bonds due to the D-A interaction, depending on the direction of charge transfer with respect to the fractional charges present on the D and A atoms. It is to be mentioned that, as the calculation of fractional charges was prohibitive at the time, Gutmann indicated electronegativity as a qualitative ground to predict them. The third rule always deals with reference to molecular species, with the necessary increase in bond lengths originating from a central atom as its coordination number is increased. Finally, the fourth rule deals with the distribution of charge among the D and A sites, stating that as a consequence of the D-A interaction, there is a local increase in electronic density at the D site and a local decrease at the A site, despite the contrary charge transfer, due to the inherent reorganization of σ bonds in the A and B molecules. In summary, bond elongation as a consequence of increased coordination number is an ordinary effect according to Gutmann’s rules, and the same effect has been described on the formation of multicenter bonds at the UTMB [5]. Thus, coordination bonds in many metal complexes can be envisaged as multicentric in nature (this being also the reason why the trans effect was initially reasoned for these compounds in inorganic and organometallic chemistry [106]).

In this context, it must be stressed that the trans influence, the increase in atomic coordination number (especially relevant in solids), and the shifting around of the electron density (piling at the donor site and spilling over at the acceptor sites), simply noted as empirical rules by Gutmann, are all now covered and rationalized in the various stages of multicenter bond formation in the UTMB [5]. As for the second rule, the qualitative electronegativity parameter has been superseded by modern methods (e.g., the ES and ET picture). This is especially needed in high-pressure studies because electronegativity is a function of pressure [98]. In this respect, we can cite here an example based on A^III^X^V^ semiconductors, e.g., GaAs, where LEP sharing (as pictured in the UTMB) and D-A interactions (as pictured by Gutmann, although now in a condensed phase and not in individual molecular partners) are equivalent processes. Lastly, the multicenter bond of the interaction was never highlighted by Gutmann, especially as the understanding of the phenomena remained at the empirical level with no bonding considerations involved [44,45]. Again, by rereading Gutmann’s rules in the light of the UTMB, one can see that molecular (B_2_H_6_, I_3_^−^, etc.) and supramolecular evidences were gathered, but not fully elucidated, nor their implications fully extended to solid phases.

As we have already commented, three-center ERMBs and EDMBs can be considered as formed by the combination of two different bonds, a primary short ionocovalent bond and a secondary long non-covalent bond. Notice that this is not the case of AX_3_ halides, where pressure-induced EDMBs are formed from primary ERMBs and secondary non-covalent bonds [17]. While this procedure of multicenter bond formation has already been mentioned in refs. [4,5], here we will present a different approach to help understand the formation of multicenter bonds not mentioned in previous works.

In relation to the above paragraph, two distinct categories of ionocovalent bonds, characterized by their dual covalent and ionic nature, can be identified. The first category encompasses bonds that represent an intermediate state between purely covalent and purely ionic interactions, commonly referred to as polar covalent bonds. Prominent examples include those found in classical binary semiconductors, such as GaAs, ZnS, and GaN. These bonds are quantitatively defined by ES < 2 and ET > 0. Graphically, they occupy a red diagonal trajectory on the ES vs. ET map (as illustrated in Figure 2), bridging the spectrum from pure covalent bonds (ES = 2, ET = 0) to pure ionic bonds (ES = 0, ET = 1). The second category comprises bonds that do not fit the description of an intermediate state. Instead, these bonds are best understood as a resonance hybrid between the covalent and ionic extremes. These are the CSBs, whose paradigmatic example is the bond in the fluorine (F_2_) molecule and other related molecules, such as bihalide and hydrogen halide molecules [6,25,57,58].

**Figure 5:**
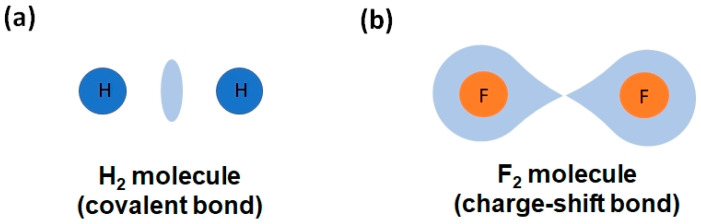
Schematic representation of isosurfaces of the electronic localization function (ELF) enclosing the attractors in the single covalent bond of the H_2_ molecule (**a**), and in the homonuclear charge-shift bond (CSB) of the F_2_ molecule (**b**). Atoms are represented by filled circles. The ELF isosurface is depicted in light blue color. In the single covalent bond of H_2_, the ELF attractor is localized at the bond critical point, the middle point between the two atoms), while in the CSB of F_2_, the ELF attractor is not localized at the bond critical point.

Unlike the case of pure single covalent bonds or polar covalent bonds, the CSB happens in interactions with a strong Pauli repulsion pressure on bonds, such as in electronegative main-group atoms, where LEPs play a significant role [6,25,57,58]. The LEP influence is especially important for halogens. A key difference between the typical covalent H–H bond in H_2_ or the covalent C–C bond of tetrahedrally coordinated C in the H_3_C–CH_3_ (ethane) molecule and the CSB of the fluorine molecule is that both show a very different electronic distribution in the region of bonding between the two atoms [6,25,57,58]. In a typical pure single covalent bond, such as the H–H bond in H_2_ (Figure 5a), there is a large ELF basin population with a small variance in the attractor occurring between the two atoms forming the bond, and the electron density exhibits a negative Laplacian at the electron density bond critical point. On the other hand, in a CSB, like that of F_2_ (Figure 5b), there is a relatively small ELF basin population with a variance as large as the population, and a positive electron density Laplacian at the bond critical point [56].

**Figure 6:**
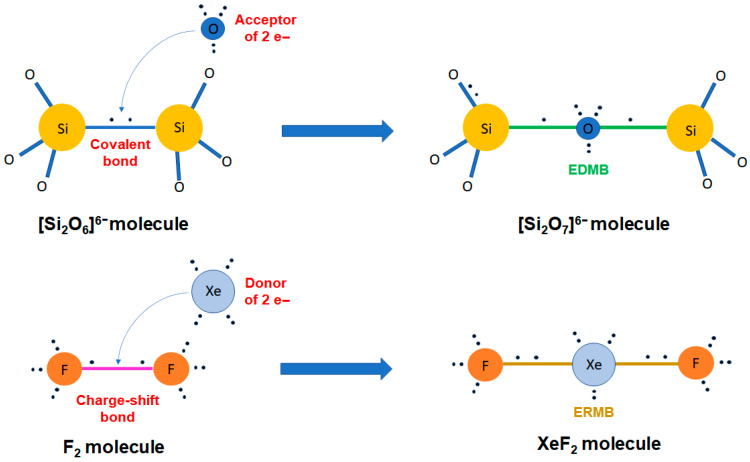
(**Top**): The linear Si–O–Si EDMB in the [Si_2_O_7_]^6−^ polyanion in Sc_2_Si_2_O_7_ can be understood as if an O atom was inserted in between the two Si atoms of a Si–Si covalent bond in a [Si_2_O_6_]^6−^ polyanion. (**Bottom**): The linear F–Xe–F ERMB in XeF_2_ can be understood as if a Xe atom was inserted in between the two F atoms of F–F CSB in the F_2_ molecule. Atoms, bonds, and electrons are represented as filled circles, solid bars, and black dots, respectively. The two-center single covalent bonds and CSBs are shown in blue and pink color, respectively, while the two-center bonds forming part of ERMBs and EDMBs are shown in gold and green color, respectively.

In this context, we want to stress that multicenter bonds, both ERMBs and EDMBs, in valence electron-rich elements/systems are made of collinear bonds, while EDMBs in valence electron-deficient elements/systems use to have angular form (see Figure 1). For example, Figure 6 top shows the linear Si–O–Si EDMB found in the [Si_2_O_7_]^6−^ polyanion of the monoclinic phase in solid Sc_2_Si_2_O_7_. This EDMB in a valence electron-rich element (O) can be understood as formed by the insertion of an O atom between two Si atoms in a Si–Si covalent bond. In this case, the O atom acts as an acceptor entity and is located at the middle of the covalent bond to complete its octet. It must be noted that Si–O covalent bonds occur in other compounds with angular Si–O–Si configurations, such as in A_2_Si_2_O_7_, (A= In, Y, La, Ce, Pr, Nd) [51], thus stressing the multicenter nature of linear or quasi-linear three-center Si–O–Si bonds. As commented previously, the presence of a 3c-2e Si–O–Si bond was already suggested by some of us not only in Sc_2_Si_2_O_7_, but also in hexamethyldisiloxane ((H_3_C)_3_-Si-O-Si-(CH_3_)_3_), and in the carbocation [(H_3_C)_3_-Si-CH_3_-Si-(CH_3_)_3_]^+^ [51,52]. Our calculations for Sc_2_Si_2_O_7_ indicated that the two equivalent Sc atoms give almost three electrons (Sc^3+^) to the [Si_2_O_7_]^6−^ anion [5]. The six electrons donated by the two Sc atoms are mainly located in the six terminal O atoms that form 2c-2e bonds with Si, while the central O atom does not receive electrons from Sc atoms. Consequently, this central O atom forms a 3c-2e bond, i.e., an EDMB formed by two interacting 2c-1e bonds, using the two electrons of the Si–Si covalent bond, while the six electrons of this O atom are located in three LEPs forming a torus around the O atom, as shown by ELF calculations. In this configuration, the central O atom that is surrounded by three LEPs acquires a trigonal bipyramidal geometry, thus forming a linear EDMB as illustrated in Figure 10a in ref. [5].

On the other hand, Figure 6 bottom shows the linear F–Xe–F ERMB in XeF_2_. In this configuration, the central Xe atom surrounded by three LEPs acquires a trigonal bipyramidal geometry forming a linear ERMB, as shown in Figure 10 in ref. [5]. This ERMB can be understood as formed by the insertion of the Xe atom between the two F atoms in the F–F CSB of the F_2_ molecule. In this case, the Xe atom acts as a donor entity and situates at the middle of the CSB so that the F atoms complete their octets. Notice that the shift of charge in the I–I–I ERMB of the I_3_^−^ anion (isoelectronic to F–Xe–F ERMB) towards the external atoms of the three-center molecule has already been discussed in our previous works [5,16]. It has been suggested that this could be a mechanism of the central atom to avoid violating the octet rule [5,107]. Alternatively, this shift of charge to the external parts of the molecule with an ERMB can be seen as the signature of the CSB present in each of the two-center bonds in a 3c-4e bond.

**Figure 7:**
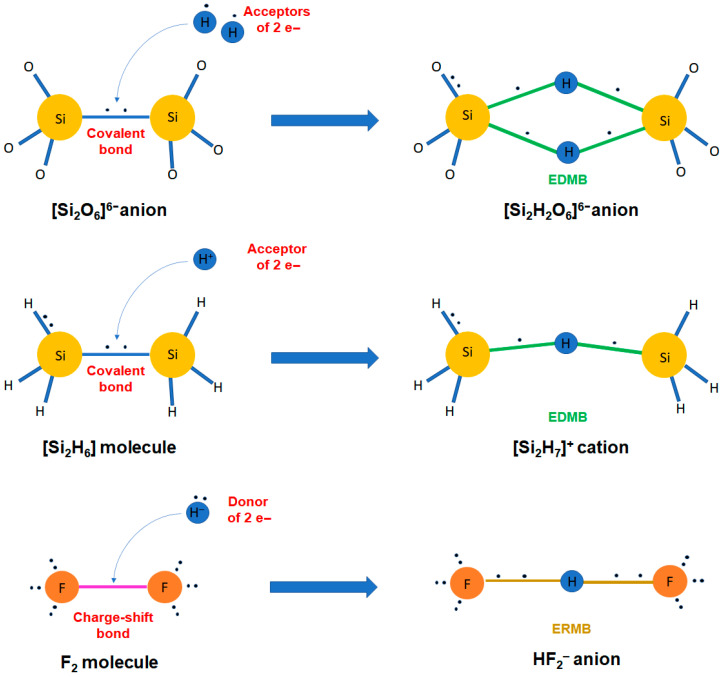
(**Top**): The triangular Si–H–Si EDMBs in the [Si_2_H_2_O_6_]^6−^ polyanion can be understood as if two H atoms were inserted in between the two Si atoms of a Si–Si covalent bond in a [Si_2_O_6_]^6−^ polyanion. (**Middle**): The quasi-linear Si–H–Si EDMBs in the [Si_2_H_7_]^+^ cation can be understood as if one H^+^ cation was inserted in between the two Si atoms of a Si–Si covalent bond in a [Si_2_H_6_] molecule. (**Bottom**): The linear F–H–F ERMB in the HF_2_^−^ anion can be understood as if a H^−^ anion was inserted in between the two F atoms of F–F CSB in the F_2_ molecule. Atoms, bonds, and electrons are represented as filled circles, solid bars, and black dots, respectively. The two-center single covalent bonds and CSBs are shown in blue and pink color, respectively, while the two-center bonds forming part of ERMBs and EDMBs are shown in gold and green color, respectively.

The acceptor O and donor Xe atoms shown in Figure 6 can be substituted by any other acceptor or donor entities to form EDMBs and ERMBs, respectively. For instance, Figure 7 top shows the two triangular (V-shape) Si–H–Si EDMBs in the [Si_2_H_2_O_6_]^6−^ anion, which can be considered to be formed by substituting the O atom in Figure 6 top by two H atoms. In this case, the two electrons of the two H atoms would add to the two electrons of the Si–Si bond to form two V-shaped 3c-2e bonds, which are similar to those present in the diborane molecule (B_2_H_6_). On the other hand, Figure 7 bottom exemplifies a linear F–H–F ERMB in the hypercoordinated HF_2_^−^ anion, which can be considered to be formed by substituting the Xe atom in Figure 6 bottom by a H^−^ anion. Another interesting case of a multicenter bond, likely a Si–H–Si EDMB, occurs in the [Si_2_H_7_]^+^ cation (Figure 7 middle) when an H^+^ cation tries to get the two electrons (to complete its doublet) between Si atoms in the [Si_2_H_6_]; a system similar to that represented in Figure 6 top. In this context, it is worthy to note that quasi-linear, symmetrical, hydride-bridged structures have been observed in [Et_3_Si–H–SiEt_3_]^+^ and [Me_3_Si–H–SiMe_3_]^+^ cations [108]. These quasi-linear bonds are similar to the quasi-linear Al-H-Al EDMB of the [Al_2_H_7_]^−^ anion, which can be considered as the insertion of an H^−^ anion between two AlH_3_ molecules [109].

**Figure 8:**
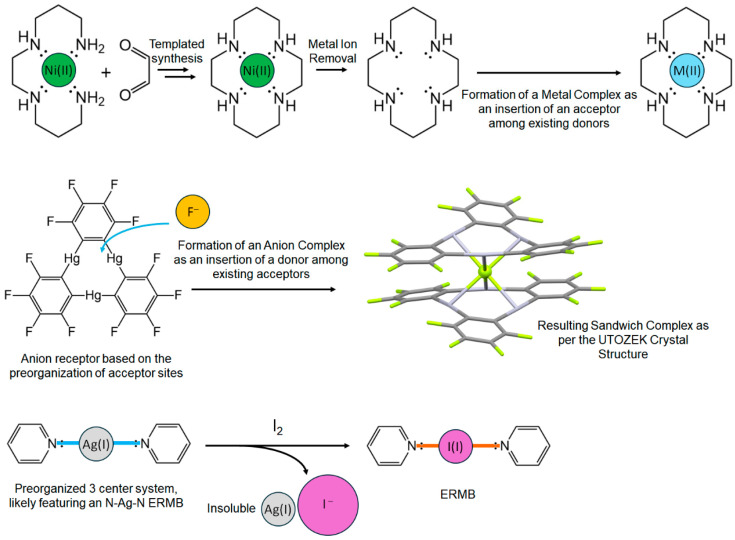
Examples of assembling molecular regions in coordination and supramolecular chemistry. Dots represent electrons. (**Top**): Formation of a metal complex by inserting acceptors on preexisting donors (Ni(II) templated synthesis of cyclam and formation of macrocyclic complexes with different metal cations). (**Middle**): Formation of an anion complex by inserting donors on preexisting acceptors (F^−^ anions inserted in Perfluoro-o-phenylenemercury). (**Bottom**): Substitution of one donor by another one (metathesis reaction) in an acceptor system (R_3_N→ ←NR_3_).

It is worth mentioning that coordination, organometallic, and supramolecular chemistry have been constructing adducts/complexes of D-A interactions relying on similar principles. This means that the formation of EDMBs and ERMBs must be very frequent in these disciplines. In this context, Figure 7 bottom could also be re-read as two preexisting F^−^ anions (8 valence electrons each) at a short distance, between which and H^+^ cation (0 valence electrons) can be formally inserted to afford the ERMB in FHF^−^ (16 valence e^−^ system). This situation is also equivalent to the situation of a proton shared between two oxyanions (or related species), R-O^−…^H^+…−^O-R, which also gives rise to ERMB and a 16 valence electrons linear system: one can plainly see how the argument is relevant to hydrogen bond and thus supramolecular affairs. As to why two anionic or donor sites should be near one another, that is frequently the case in preorganized ligands for metal cations. Coordination chemistry has mostly worked by assembling molecular regions lacking bonds where atoms/ions could logically be inserted (Figure 8). As an example, one could refer to the templated synthesis of macrocycles and cages. A classical case would be Ni(II)-templated synthesis of cyclam followed by metal ion removal (e.g., cyanidation) [110]. At that point, the act of forming a metal complex using the so-prepared ligand is formally equivalent to the insertion of a metal cation in between the preexisting and prearranged (although non-directly bonded) donor atoms (Figure 8 top). When the whole process is considered, it can be viewed as a transmetalation reaction in which the central cation is swapped. This common paradigm for cation receptors has been reversed in preparing receptors for anions, where a cluster of acceptors is assembled so that a donor can be inserted into it (Figure 8 middle). For instance, the UTOZEK crystal structure is formed by inserting an F^−^ anion into acceptor Perfluoro-*o*-phenylenemercury with an Hg_3_ anticrown site [111].

Similar ideas have long been exploited in order to successfully achieve stereoelectronic complementarity, a pillar concept in coordination and supramolecular chemistry. While multicenter bonds do not necessarily form in all such cases (it depends on the valence electron count), there are recent clear examples of assemblies achieved by first preassembling the donors and then swapping a central classical acceptor (metal cation) with a non-classical acceptor. For example, linear Ag^+^ complexes with N donor atoms (N: − Ag^+^− :N) have been prepared by Rissanen and coworkers as precursors to their I(I) analogues (Figure 8 bottom). The Ag(I) complexes have then been exposed to I_2_ to generate Ag^+^I^−^ (insoluble) and to insert the in situ generated I^+^ cation in the preexisting (N: → ← :N) system, forming the N: − I^+^− :N complex [112]. The R_3_N−I−NR_3_ central moiety has a 22 e^−^ valence count (as I_3_^−^) and is now kept together by an undeniable 3c-4e ERMB.

The above examples allow us to point out that insertion/incorporation/swapping of central atoms in linear systems is not only a convenient way of depicting the matter but is also closer to established concepts and synthetic protocols than it may appear at first.

At this point, we want to stress the strong relationship between ERMBs and CSBs since the CSB, which is present in each of the individual F–Xe and Xe–F bonds of the F–Xe–F ERMB in the hypercoordinated XeF_2_ molecule and other hypercoordinated molecules, has already been discussed by Braïda and Hiberty [70,71]. The similarity of the chemical bonding in CSBs and ERMBs is evidenced by the fact that bonds in molecules with these two bond types show similar ES and ET values for their constitutive two-center bonds (note that ES and ET values can only be calculated for two-center bonds). Therefore, each of the two-center bonds of the three-center bond is located in the same region (orange) of the ES vs. ET map; the region we previously considered occupied by ERMBs (see Figure 2) [5]. Additional examples of CSBs that lie in the region of ERMBs are hydrogen halide (HF, HCl, HBr, HI) and dihalide (F_2_, ClF, BrF, and IF) molecules (see Table 1). Calculations of these molecules were carried out using the Quantum ESPRESSO package [113], and the subsequent analyses (ES, ET, as well as Bader and Löwdin charges) were performed using the CRITIC2 and LOBSTER codes, as already explained in previous works [4,5,10,11,12,13,14,15,17,19]. The ES and ET values of these CSBs can be compared to molecules with ERMBs, such as XeF_2_, I_3_^−^, and HF_2_^−^, either in the ES vs. ET map obtained from density-based or from orbital-based calculations [4,5,20]. In addition to the two-center bonds in the F–Xe–F ERMB of XeF_2_, in the I–I–I ERMB of I_3_^−^, and in the F–H–F ERMB in HF_2_^−^, the ES vs. ET map also shows the location of the two-center bonds in the B–H–B EDMB in B_2_H_6_ and Si–O–Si EDMB in Sc_2_Si_2_O_7_ (see data of these last compounds in ref. [5]).

In conclusion, CSBs and ERMBs can be considered as bonds formed by the resonance of covalent and ionic interactions extended to two and three centers, respectively. The difference is that ERMBs are multicenter bonds, in fact three-center bonds, due to the electron delocalization along three centers, while CSBs, being two-center bonds, do not show a multicenter character due to the inherent electron localization between the two centers. Note that these “resonant-like” bonds have nothing to do with the resonant delocalized covalent bonds, which are typically a mixture (resonance) of single and double covalent bonds. We also want to point out that, to the best of our knowledge, no linear molecules longer than three centers and with CSB between each two centers have been reported yet [57,58]. This finding supports the claim of the UTMB that ERMBs (alone) cannot be found in linear molecules longer than three centers [5]. In summary, ERMBs are formed by two collinear CSBs and can be considered as the insertion of a donor atom with low electronegativity between two highly electronegative atoms, which can be considered to feature a (two-center) CSB.

The formation of CSBs and ERMBs (two types of ionocovalent resonant bonding) can be understood by considering that these are usually formed by very electronegative atoms (e.g., halides). These atoms are so eager for electrons that they can hardly share their valence electrons; i.e., they hardly form covalent bonds because they just want to bond to any atom that donates them the electron they need to complete the octet. Therefore, the links between these highly electronegative atoms can be seen as a “fight” to catch all available bonding electrons, which are not shared (as in the H_2_ molecule) but stolen from the neighboring atom. When viewed in a static way, we can consider that the resonant bond results in an “average” sharing of electrons; however, in a dynamic view, it can be understood as an alternate theft of electrons from one atom to another since both atoms are highly electronegative. In other words, we can see this bond as a “dynamic” motion of electrons between two highly electronegative atoms, and this is the reason why there is not a high electron density in the bond critical point of F_2_, unlike in the H_2_ molecule. This alternative theft of electrons applied to the CSB in the F_2_ molecule allows us to understand that this bonding cannot be seen as an actual sharing of electrons between the two F atoms (as in a covalent F–F bond), but mainly as a combination of F^+^–F^−^ and F^−^–F^+^ moieties in the wavefunction. It is interesting to notice that the F^−^ ion is a pseudo-Ne (ψ-Ne) atom, which has negligible electronegativity (closed shell), while an F^+^ ion is a ψ-O atom, which is highly electronegative. In this way, it can be understood that the ψ-O atom tends to steal two electrons from the ψ-Ne atom. Once the ψ-O atom takes the two electrons from the ψ-Ne atom, the ψ-O atom becomes a ψ-Ne atom and the ψ-Ne atom becomes a ψ-O atom, thus reversing the process, so that this process of theft of electrons goes on and on indefinitely. This seems to be characteristic of CSBs.

To conclude this section, we want to mention that the two-dimensional map of shared versus transferred electrons (ES vs. ET), in which the different types of bonds can be allocated (Figure 2), serves as a powerful diagnostic tool for classifying chemical bonds within the framework of the UTMB. Its versatility allows us to plot even resonant bonds (purple region), which occupy a distinct region of the map characterized by ES > 2. This region encompasses not only conventional double and triple covalent bonds, such as those in O_2_ and N_2_, respectively, but also delocalized resonant bonds (intermediate between single, double, and triple bonds), such as those in benzene and in the graphene layers of graphite. It is curious that all “resonant” bonds (resonant delocalized, CSB, and ERMB) occupy the same region of the ES vs. ET map; i.e., the region above the covalent and ionic bonds. In summary, the ES vs. ET map provides a unified view of most (if not all) primary chemical bonds in molecules and solids that can be used in all fields of chemistry and materials science.

The methodology that has to be followed to calculate both ET and ES values is crucial to accurately position bonds on the ES vs. ET map. The ES value can be easily calculated as twice the delocalization index (in density-based methods, such as CRITIC2) or as twice the integrated crystal orbital bond index (in orbital-based methods, such as LOBSTER). The ET value instead is generally derived from atomic charges, such as Bader’s charges in density-based methods or Löwdin/Mulliken charges in orbital-based methods. However, the procedure has to consider the ionicity of the bond. In simple cases of binary compounds (e.g., GeTe), the ET value can be calculated as the cation charge divided by the nominal oxidation state [5]. In more complex cases, such as ternary compounds containing polyanions (e.g., CsI_3_ and Cs_2_TeI_6_), a more nuanced approach is necessary. To determine ET for the I–I bond in the I_3_^−^ polyanion or the Te–I bond in the [TeI_6_]^2−^ polyanion, one has to calculate the difference in atomic charges between the two bonded atoms. This ET value considers the real ionicity of the bond and not only the electrons transferred from I to I in the I_3_^−^ polyanion or from Te to I in the [TeI_6_]^2−^ polyanion. In other words, to calculate ET, one has to account for the electrons already transferred from Cs atoms to the polyanions. In summary, the ES vs. ET map, taken within the UTMB, can be considered a “treasure map”, since we can distribute the different types of known “two-center bonds” and trace the change in chemical bonding between any two atoms under varying circumstances, even though the two-center bonds can be part of multicenter bonds.

## 6. Conclusions

In this work, we have introduced the unified theory of multicenter bonding (UTMB) and commented on the main classical (resonant, covalent, ionic, metallic) and exotic (EDMB, ERMB, and CSB) types of bonding in materials and how they have been integrated in the UTMB. The UTMB makes use of the ES vs. ET map to illustrate that the different chemical bonds can be typically found in different regions in this revisited version of the covalency vs. ionicity map to obtain an integral and unified view of chemical bonding, the heartland of chemistry, in molecules and solids across all disciplines.

We have shown that the UTMB is a daughter of high-pressure studies in materials and shows the origin and formation mechanism of multicenter bonds; a matter of debate for over 70 years due to the unbalanced research of chemical bonding in molecules and solids. The UTMB is based on the number of electrons shared between any two atoms (non-bonding electrons in lone electron pairs (LEPs) play a negligible role in bonding except in CSBs), on the doublet/octet rule (generally verified even in early assumed “hypervalent” molecules), and the VSEPR theory (that governs the disposition of electrons around an atom to minimize Pauli electronic repulsion).

The UTMB emphasizes that the formation of multicenter bonds occurs due to the electron delocalization in hypercoordinated atoms and is favored for high electronic density conditions, such as those provided at high pressures, when atoms in a group are substituted by their heavier analogs, and when systems are reduced (contrary to oxidation), i.e., when electrons are inserted in the system.

We have commented that our initial efforts of application of the UTMB have been directed to demonstrate that EDMBs can be observed in electron-rich elements, thus providing a unified view of ERMBs and EDMBs in electron-rich systems, unlike previously assumed; however, the UTMB can be applied to any compound, since it provides a more comprehensive understanding of chemical bonding than current theories. This is achieved by placing the two multicenter bonds, and now also CSBs, on equal footing as classical resonant, covalent, ionic, and metallic bonds, as observed in the ES vs. ET map. This has resulted in a revised van Arkel–Ketelaar diagram (see Figure 3b).

The simple rules established in the UTMB are general and have allowed us to explain the unknown multicenter bonds present in very different materials. Among others, these include the two known phases of polonium at room pressure; the high-pressure sixfold-coordinated phases of pnictogens and chalcogens [4]; the crystalline phases of PCMs (SnTe, PbS, PbSe, and PbTe) [9,10], and related ternary compounds, such as PbBi_2_Te_4_, at room pressure [11]; several Zintl compounds (Li_2_Sb, BaZnSb_2_, Cs_2_Te_5_, Cs_2_TeI_6_, and TlTe) [5]; metal iodates [12,13]; Rashba semiconductors, such as BiTeX (X = Cl, Br, I) at room pressure [14]; AIO_3_ perovskites (RbIO_3_ and CsIO_3_) [15]; and even metals with *p*-type electrons [19].

Moreover, the UTMB has allowed us to propose (for the first time) a mechanism of formation of the perovskite structure in BX_3_ and ABX_3_ compounds as the result of a polymerization process of the BX_3_ units [15]; to explain the different geometries experimentally found in polyiodides, an issue for more than 50 years [16], to show the transformation of ERMBs into EDMBs under compression in AX_3_ halides [17]; and to account for the chemical bonding present in an infinite linear chain of H and I atoms [5,17,18]. Therefore, we can conclude that the formation of multicenter bonds is behind many polymerization processes. Finally, the UTMB has challenged the current understanding of metallic bonding since a multicenter character along certain directions has recently been discovered in the metallic bonds of elements where *p*-type electrons participate in bonding, like in the metallic phases of group-14 elements [19]. In this context, the UTMB has shown us that many collinear bonds, previously assumed to be somewhat deficient covalent bonds, are indeed multicenter bonds. A situation that is likely to be found many times in coordination, organometallic and supramolecular chemistry. In fact, we have recently been awared that 3c-2e EDMBs and 3c-4e ERMBs in organometallic chemistry are referred as (or related to) agostic and anagostic interactions, respectively [114], thus showing the proliferation of different notations of similar concepts in different fields of chemistry that prompt a revision of IUPAC under the light of the UTMB.

Our findings make us confident that UTMB will likely allow us to tailor the properties of materials of interest for relevant technological applications, in addition to the value it brings regarding basic research on materials. In this respect, we need to emphasize that ERMBs typically lead to insulating or semiconducting materials, while EDMBs typically provide materials with semiconducting and semimetallic properties; e.g., CsI_3_ is an insulating material with ionic Cs–I_3_ bonds and I–I ERMBs within I_3_^−^ units [18]; Cs_2_Te_5_ is a narrow-gap semiconductor with ERMBs [115]; CsPbI_3_ is a semiconductor with Cs–I ionic bonds and Pb–I EDMBs forming infinite linear chains along the three directions of space of the cubic perovskite structure; and tetragonal CsIO_3_ is a semimetal with Cs–O ionic bonds and I–O EDMBs forming infinite linear chains along two crystallographic directions of the tetragonal perovskite structure [15].

All in all, we hope that the present introduction to the UTMB, extended with the inclusion of the CSB, will entice all materials researchers (including organic, inorganic, solid-state, coordination, organometallic, and supramolecular chemists) to study the UTMB and apply it in their respective domains, to get a deeper understanding of material properties (including molecules and solids) beyond previous assumptions and to develop enhanced technological applications for a greener future.

## Figures and Tables

**Table 1:** Calculated quantum-chemical bonding descriptors for selected molecular systems featuring CSBs by using the density-based approach, as implemented in the CRITIC2 (version 1.2) software, and the orbital-based method, as implemented in the LOBSTER (version 5.1.0) program. The Bader atomic charges, the renormalized number of electrons transferred between two atoms (ET), and the number of electrons shared between each atom pair (ES) are provided, as well as the bond distances and the bond types. The ES is defined as 2 × DI, where DI is the delocalization index between two atoms, as calculated by CRITIC2, while it is defined as 2 × ICOBI(2c), where ICOBI(2c) is the integrated crystal orbital bond index for two centers, as calculated by LOBSTER.

Species	Bader Charge		ET Bader Charge	ES (2 × DI)	Löwdin Charge	ET Löwdin Charge	ES (2 × ICOBI(2c))	Bond Length (Å)	Bond Type
HF	H	+0.726	0.726	0.98	+0.51	0.51	1.51	0.992	CSB
	F	−0.726			−0.51				
HCl	H	+0.266	0.266	2.02	+0.21	0.21	1.91	1.281	CSB
	Cl	−0.266			−0.21				
HI	H	+0.080	0.080	2.38	+0.02	0.02	2.00	1.614	CSB
	I	−0.080			−0.02				
F_2_	F	0	0	2.47	0	0	2.15	1.397	CSB
ClF	Cl	+0.406	0.406	2.52	+0.34	0.34	1.77	1.639	CSB
	F	−0.406			−0.34				
BrF	Br	+0.493	0.493	2.35	+0.39	0.39	1.69	1.772	CSB
	F	−0.493			−0.39				
IF	I	+0.596	0.596	2.15	+0.50	0.5	1.5	1.920	CSB
	F	−0.596			−0.50				

## Data Availability

The raw data supporting the conclusions of this article will be made available by the authors on request.
